# Microbiota Associated with Different Developmental Stages of the Dry Rot Fungus *Serpula lacrymans*

**DOI:** 10.3390/jof7050354

**Published:** 2021-04-30

**Authors:** Julia Embacher, Sigrid Neuhauser, Susanne Zeilinger, Martin Kirchmair

**Affiliations:** Department of Microbiology, University of Innsbruck, Technikerstrasse 25, 6020 Innsbruck, Austria; julia.embacher@uibk.ac.at (J.E.); sigrid.neuhauser@uibk.ac.at (S.N.); susanne.zeilinger@uibk.ac.at (S.Z.)

**Keywords:** bacterial community, wood-decaying fungi, *Serpula lacrymans*, microbiota, bacterial–fungal interactions, fungi/bacteria of the built environment

## Abstract

The dry rot fungus *Serpula lacrymans* causes significant structural damage by decaying construction timber, resulting in costly restoration procedures. Dry rot fungi decompose cellulose and hemicellulose and are often accompanied by a succession of bacteria and other fungi. Bacterial–fungal interactions (BFI) have a considerable impact on all the partners, ranging from antagonistic to beneficial relationships. Using a cultivation-based approach, we show that *S. lacrymans* has many co-existing, mainly Gram-positive, bacteria and demonstrate differences in the communities associated with distinct fungal parts. Bacteria isolated from the fruiting bodies and mycelia were dominated by Firmicutes, while bacteria isolated from rhizomorphs were dominated by Proteobacteria. Actinobacteria and Bacteroidetes were less abundant. Fluorescence in situ hybridization (FISH) analysis revealed that bacteria were not present biofilm-like, but occurred as independent cells scattered across and within tissues, sometimes also attached to fungal spores. In co-culture, some bacterial isolates caused growth inhibition of *S. lacrymans,* and vice versa, and some induced fungal pigment production. It was found that 25% of the isolates could degrade pectin, 43% xylan, 17% carboxymethylcellulose, and 66% were able to depolymerize starch. Our results provide first insights for a better understanding of the holobiont *S. lacrymans* and give hints that bacteria influence the behavior of *S. lacrymans* in culture.

## 1. Introduction

**Figure 1:**
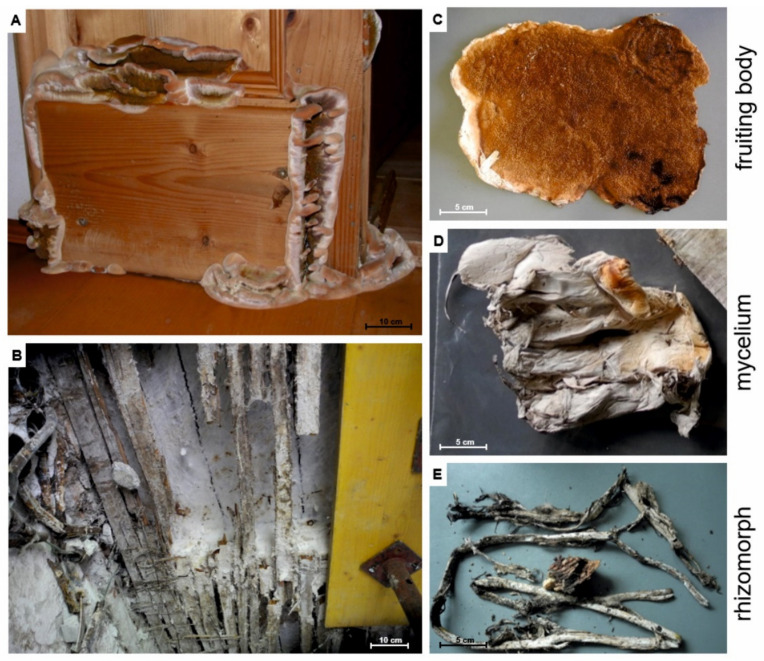
Infestation of timber by the dry rot fungus *Serpula lacrymans* and tissue types of *S. lacrymans*. (**A**) Damaged wood with fruiting body; (**B**) fungal mycelia on a wood beam ceiling; (**C**) fruiting body; (**D**) aerial mycelium; and (**E**) rhizomorph (cord mycelium) with wood.

*Serpula lacrymans* is a very effective brown rot-causing fungus, specialized in the degradation of coniferous timber in buildings. The initial colonization is favored by water damage, and after establishment the fungus starts to degrade cellulose and hemicellulose (Figure 1A,B). It is among the most feared wood-rotting fungi in the built environment as the remediation of *S. lacrymans*-damaged buildings is expensive and tedious. After improper renovation, the possibility of recolonization by *S. lacrymans* is likely. Different tissue types of *S. lacrymans* include the fruiting body (Figure 1C), aerial mycelia (Figure 1D) and rhizomorph-like transport organs (“rhizomorphs”—Figure 1E). The colonization of wood by *S. lacrymans* is characterized by the rapid growth of vegetative mycelia and the formation of thick (up to 2 cm diameter, Figure 1E) mycelial cords, which are used to transport nutrients and water to the hyphae exploring new wood substrates (here also called “rhizomorphs”) [1]. This allows *S. lacrymans* to rapidly grow and makes it a successful invader in built environments [1,2], resulting in particular economic importance of dry rot.

The dry rot fungus colonizes wood/timber in buildings especially after water damage [3]. The establishment can be influenced by bacteria, which are able to alter the wood permeability and structure by the production of, e.g., cellulases and pectinases, which open the crystalline structure of the cellulose microfibrils [4], thereby increasing the accessibility of the wood components for decay by fungi. The colonization of wet wood by bacteria generally happens at the beginning of the decay process [5,6]. The bacteria enter dead wood or timber via the xylem parenchyma cells of ray tissues, vessels, tracheids and other wood cells, mainly using the pits in cell walls for their penetration. There, they benefit from the degrading progress by living on pectin, monosaccharides and other easily accessible nutrients [7].

It was shown that wood-decomposing bacteria live in close interaction with fungi [5,8,9,10,11], and that those bacteria are able to process low-molecular-mass sugars and small aromatic compounds that are released by lignocellulolytic fungi [12,13]. It is hypothesized that there is a synergistic interaction between bacteria and soft-rot fungi to predispose wood to degradation by other fungi [5], and it was discussed that the cellulase producer *Trichoderma viride* plays a role in timber degradation together with basidiomycetous wood-rotting fungi [14,15]. Timber degradation, hence, is likely a process to which different organisms are participating that inter-, co- and counteract with each other.

Nonetheless, fungi are considered as superior wood decomposers because of their larger size and mobility (via hyphal growth), and their distinguished enzymatic, as well as non-enzymatic, capability to degrade the mayor building blocks of wood (lignin, cellulose, and hemicellulose) [5,13,16]. It is well established that the initial stages of dry rot involve the non-enzymatic action of highly destructive hydroxyl radicals produced in a process called Fenton chemistry. Fungal hyphae secrete oxalic acid, which forms complexes with Fe^3+^, low-molecular-weight (LMW) iron-reducing compounds, and hydrogen peroxide (H_2_O_2_), which diffuse into the wooden cell wall. There, LMW compounds sequester Fe^3+^ from the Fe–oxalate complexes and reduce it to Fe^2+^. Fe^2+^ can react with H_2_O_2_, which is the actual Fenton reaction, and generate hydroxyl radicals (•OH). Upon attack of OH radicals, the lignocellulose matrix is disrupted [17].

During wood degradation by basidiomycetes, the environmental conditions become very challenging for bacteria because of, e.g., toxic fungal secondary metabolites [18]. Bacteria are not only faced with low-pH and highly destructive radicals during dry rot decay, but also with Ca^2+^ and Fe ^3+^ chelators like oxalic acid, which sequester all available anions [17]. In *Picea abies* logs, the fungal diversity correlated negatively with the bacterial abundance, while certain bacterial taxa co-occurred with certain fungi [19]. The colonization of beech woodblocks with the white-rot fungi *Hypholoma fasciculare* and *Resinicium bicolor* resulted in a strong bactericidal effect [20]. The structure of wood-associated bacterial communities was altered by the white-rot *Phanerochaete chrysosporium* [21], while in soil, basidiomycetes appeared to affect the community composition of bacteria [22,23]. Basidiomycete-associated bacteria need to survive these harsh conditions [18]; hence, bacteria must have evolved strategies to utilize fungal-secreted metabolites and overcome fungal defense mechanisms [24].

Bacterial–fungal interactions (BFI) can have different levels of specificity and a diverse range of interactions from antagonistic to beneficial relationships [2,5,6,22,25,26,27]. This means that the co-occurrence of bacteria and fungi is a result of physiological and metabolic interactions during which bacteria and fungi co-evolve and interdependently evolve. However, co-occurrence may be accidental and not representative of any causal relationship but the result of stochastic ‘mixing’. Any interaction between fungi and bacteria can modulate the behavior of neither, one or both of the interaction partners [28]. Such BFI include competition for substrates [20] or the production of growth factors for fungi [5]. The carbon-to-nitrogen ratio in wood is low and an increase in nitrogen by nitrogen-fixing bacteria living at the cost of carbohydrates set free by the fungi may be important [29,30]. Hence, the assemblage of bacteria that surround and interact with a fungus effectively constitute its microbiome, and as such, they must be considered together [26]. Therefore, the term holobiont is useful, as it is defined as a “unit of biological organization composed of several distinct genomes, that, in principle, influence the genomic evolution of each other” [28,31].

Only little is known about the natural bacterial and fungal interaction partners of *S. lacrymans*, although there are numerous fungal microbiome studies available [32,33,34,35,36]. So far, the analysis of the airborne fungal spores in *S. lacrymans*-damaged homes revealed the co-occurrence of the ligninolytic *Donkioporia expansa* and the mycoparasite *T. viride* [14]. Otherwise, many studies indicated that communication between interacting partners is key for any interaction. The communication molecules for bacteria and fungi include antibiotics and other secondary metabolites (SMs) produced by both partners, which may be involved in mutualism, chemical warfare and in signaling [37]. Fungus-associated bacteria have been shown to affect the secondary metabolism of various fungi [38,39,40,41,42]. A recent study analyzed the effect of bacteria on *S. lacrymans* pigment production [43,44]. Pigment synthesis from the quinone precursor atromentin, such as variegatic acid, was stimulated by 13 different bacteria and cell wall-lysing enzymes (e.g., β-glucanase, cellulase, proteases and chitinases), but not by lysozyme or mechanical damage. This may indicate a common pigmentation-inducing mechanism, which could be triggered by fungal cell wall degradation. Moreover, the fungal pigments variegatic and xerocomic acid impact biofilm expansion and the swarming motility of bacteria such as *Bacillus subtilis* [44]. During co-culturing with ubiquitous bacteria, the gene cluster encoding for a synthetase and aminotransferase, both needed for atromentin biosynthesis, was induced [45].

As bacterial–fungal interactions can influence fungal establishment on wood, the aim of this study was to characterize the bacterial community associated with *S. lacrymans* using culture-based and microscopic approaches. The bacterial isolates were characterized using physiological tests and via bacterial–fungal interactions. This allowed a better understanding of their potential influence on *S. lacrymans.* The focus was on investigating the spatial abundance, composition and properties of the bacterial community of different *S. lacrymans* tissue types, including fruiting bodies, mycelia and rhizomorphs. The different developmental stages have different metabolic profiles and we wanted to understand (i) if there is a tissue type-specific community composition and (ii) if interaction partners change the behavior of *S. lacrymans* during co-culture experiments. We addressed these questions by isolating the bacteria associated with different *S. lacrymans* tissues and the subsequent establishment of 16S rRNA gene fingerprints. Microscopic analyses (fluorescence in situ hybridization (FISH)) were used to obtain information about the spatial distribution of bacteria in different *S. lacrymans* tissues. Furthermore, the biopolymer degradation properties of the bacterial isolates were determined. Overall, *S. lacrymans* has numerous bacterial partners, most of them Gram positive. The obtained results provide first evidence that different tissue types are preferentially colonized by different bacterial phyla. In co-culture assays, few bacterial isolates caused growth inhibition of *S. lacrymans,* and vice versa, while many of the interactions remained neutral in our test system.

## 2. Materials and Methods

### 2.1. Biological Material

*Serpula lacrymans* fruiting bodies, mycelia and rhizomorphs were collected indoor from infested timber in Austria, between summer 2018 and November 2019 (Table 1). The samples were collected, transferred into fresh zip-loc plastic bags and stored at 4 °C until processing (not more than 48 h). These fresh samples were used to isolate bacteria, for assessing bacterial CFU g^−1^ tissue and for FISH analysis as described below. To gain *S. lacrymans* pure cultures, a fruiting body from Innsbruck, collected in autumn 2018 (no. 1SLIBK2018; NCBI ITS sequence is available under no. MW491273), was cut with a sterile scalpel and material from the inside of the fruiting body was placed on malt extract agar (MEA, per l: 30 g malt extract, 3 g soy peptone, 15 g agar; all from Roth, Karlsruhe, Germany). The plates were incubated at 25 °C for 1 to 2 weeks. The fruiting body for gaining the fungal pure culture was not used for bacterial isolation.

### 2.2. Isolation of Bacteria from S. lacrymans Fruiting Bodies, Mycelia and Rhizomorphs

The fruiting bodies, mycelia and rhizomorphs for bacterial isolation were collected in the period from summer 2018 to November 2019 (Table 1, no. 1–18, Figure 1C fruiting body, Figure 1D mycelia, Figure 1E rhizomorph). The fungal tissue (0.1–1 g, depending on the sample type) was incubated in 20 mL 0.9% sodium chloride solution containing 0.015% Tween^®^ 80 (Sigma-Aldrich, St. Louis, MO, USA) with 10 sterilized glass beads (Ø 4 mm) for 10 min using a rotary shaker. Decimal dilution series up to 10^−6^ were prepared from this solution. Volumes of 500 µL or 100 µL of each dilution step were plated on MEA plates, tryptone soy agar (TSA, per l: 15 g casein peptone, 5 g soy peptone, 5 g NaCl, and 15 g agar; all from Roth, Karlsruhe, Germany), and Roth’s R2A agar (per l: 0.5 g yeast extract, 0.5 g peptone, 0.5 g casein hydrolysate, 0.5 g glucose, 0.5 g starch, 0.3 g KH_2_PO_4_, 0.04 g MgSO_4_, 0.3 g sodium pyruvate, and 15 g agar), and incubated at 25 °C for two to seven days. Plates were checked daily for growth of bacteria. The samples with no. 7–15 and 17 (Table 1) were used to estimate the total CFU g^−1^ tissue.

To randomly isolate the bacterial colonies, each petri dish was divided into grids of 5 mm × 5 mm (264 boxes in total). The boxes were numbered and random numbers were drawn to choose the isolates. All the isolates were dilution plated three times before further processing. Using an inverse morphotype approach (Figure S1), abundances of bacteria isolates (in CFU g^−1^) were estimated by counting similar morphotypes. Based on 16S rRNA gene fingerprints, the colonies of similar morphology were assigned to the same group. Only the plates that contained a certain phylotype were included in the inverse morphotype assay. The isolates that had a highly similar morphotype could not be distinguished. Their abundance was estimated via the proportion of sequenced isolates within the individual samples. Therefore, it is important to stress that all taxonomic assignments of bacterial species are an approximation.

### 2.3. Characterization of Isolated Bacteria

#### 2.3.1. Molecular Identification and Phylogenetic Placement

We established fingerprints of the microbiota on the basis of 16S rRNA gene sequencing. Therefore, bacterial colony PCR was performed with the Red Taq 2x DNA Polymerase Master Mix (VWR, Radnor, USA; TRIS-HCl pH 8.5, (NH_4_)_2_SO_4_, 4 mM MgCl_2_, 0.2% Tween^®^ 20, each time 0.4 mM dNTPs, 0.2 units µL^−1^ Taq Polymerase, inert color reagent). The PCR mixture contained 10 µmol of each of the primers 27F (5′-AGA GTT TGA TCA TGG CTC A-3′) and 1492R (5′-TAC GGT TAC CTT GTT ACG ACT T-3′), 10.75 µl distilled water and 0.5 µL bovine serum albumin (BSA, 2%). The bacterial single colonies, not older than 3 days, were picked with a toothpick and added to the PCR mixture. The PCR conditions were 95 °C for 10 min, 30 cycles of 95 °C for 30 s, 53 °C for 30 s, 72 °C for 45 s, and a final elongation step at 72 °C for 10 min. The PCR products were subject to agarose gel electrophoresis (100 V, about 20 min) to confirm their correct size and they were subsequently sequenced using the Microsynth sequencing service (Balgach, Switzerland). The sequences were manually aligned using the BioEdit sequence alignment editor version 7.0.5.3 [46]. The reference sequences were retrieved from the NCBI database. Phylogenetic trees were generated using PhyML (version 3.2, www.atgc-montpellier.fr/phyml) with implemented Geneious version 9.1.8 and a general time-reversible (GTR) substitution model and Chi2 statistics to estimate branch support. The trees were optimized using the topology/length/rate function using NNI (next neighbor interchange) to search for the optimal tree topology. For phylogenetic placement Chi^2^ was used because it is a fast method and absolutely suitable for our aim to create a similarity matrix instead of a phylogenetic tree. The sequences are available from the NCBI sequence read archive with the accession numbers MW089011 to MW089305 (for details see Table S1 in the supplement). The isolates were assigned a genus (e.g., *Bacillus* sp.) and comparative literature searches were conducted referencing the closest related species.

#### 2.3.2. Biopolymer Degradation Tests and Evaluation

The bacterial isolates were tested for enzymatic activities that might be useful in the dead wood environment, such as, xylanases, cellulases, pectinases, and amylases [47]. The bacteria were streaked out without standardization and incubated at 25 °C until bacterial colonies were visible. Up to six bacteria were streaked on one plate. These qualitative tests were performed once per isolate.

Starch agar (per l: 10 g potato starch, 5 g pancreatic digest of gelatin, 3 g beef extract, and 15 g agar; pH 6.8 ± 0.2; all from Roth, Karlsruhe, Germany) was used for the detection of starch-hydrolyzing microorganisms [48]. The inoculated plates were incubated at 25 °C for 48 to 96 h.

Screening for pectinase activity was performed on pectinase screening agar (PSAM; per l: 2 g NaNO_3_ (Merck, Darmstadt, Germany), 0.5 g KCl (Roth), 0.5 g MgSO_4_ (Roth), 1 g K_2_HPO_4_ (Roth), 0.5 g tryptone (Roth), 20 g agar (Roth), and 10 g pectin (Sigma, St. Louis, MO, USA); pH 5.5 ± 0.5) [49]. The inoculated plates were incubated at 25 °C for 48 to 96 h.

Cellulolytic activity was assessed on carboxymethylcellulose agar (CMC agar; per l: 10 g peptone (Roth), 10 g carboxymethylcellulose (Fluka, St. Louis, MO, USA), 2 g K_2_HPO_4_ (Roth), 0.3 g MgSO_4_·7H_2_O (Roth), 2.5 g (NH_4_)_2_SO_4_ (VWR, Radnor, PA, USA), 2 g gelatin (Gatt-Koller, Absam, Austria), and 15 g agar (Roth); pH 6.8 ± 0.2 [50]). The inoculated plates were incubated at 25 °C for 48 to 96 h.

Screening for xylanase activity was done using minimal agar medium (per l: 0.05 g MgSO_4_·7H_2_O (Roth), 0.05 g NaCl (Roth), 0.01 g CaCl_2_ (Sigma, St. Louis, MO, USA), 0.2 g yeast extract (Roth), 0.5 g peptone (Roth), and 15 g agar (Roth); pH 6.8 ± 0.2) [51], with 0.5% oat-spelt xylan (Serva, Heidelberg, Germany) as the only carbon source [52]. The inoculated plates were incubated at 25 °C for 48 to 96 h.

After incubation, all biopolymer-degradation test plates were flooded with approximately 10 mL of 50 mM potassium iodide–iodine solution (Merck, Darmstadt, Germany) and incubated for 15 min at room temperature. The excess iodine solution was poured off and the plates were washed with 1 M NaCl [48]. Another tested evaluation method was the flooding with 0.1% (*w*/*v*) Congo red solution (Fluka, St. Louis, MO, USA) followed by washing with 1 M NaCl after 15 min [53]. Both the evaluation methods worked equally well. The formation of a clear hydrolysis zone around the bacterial colonies indicated enzymatic activity. The tested isolates were allocated to one of the following four classes: no production of according enzyme (-, no halo visible), minimal production of enzyme (~, halo zone of no more than 1 mm from the edges of the colonies), enzymatic activity (+, halo zone smaller than 8 mm from the edges of the colonies), and high enzymatic activity (++, halo zone larger than 8 mm).

#### 2.3.3. Co-Culture of *S. lacrymans* with Isolated Bacteria

Co-cultures were performed on 85 mm petri dishes containing 20 mL minimal media (MM) with a low nitrogen-to-carbon ratio (C:N = 400:1) similar to the values found in wood [54,55]. To prepare the bacterial inoculum for the co-cultivation experiments, the bacterial strains were incubated at 25 °C for up to 7 days on MM before transferring them onto plates containing the *S. lacrymans* strain (no. 1SLIBK2018). 1SLIBK2018 had been pre-grown for three weeks on MM agar plates at 25 °C, before transferring it to the center of the confrontation plate where it was allowed to grow for seven days at 25 °C before the bacteria were added (Table 2). In total, 50 bacterial isolates across the taxonomic range were tested. The 50 isolates were chosen based on their taxonomic position to cover the full taxonomic range of isolates rather than focusing on specific abundantly isolated groups. The co-culture plates were incubated at 25 °C for 72 days. For the first 14 days, the plates were photographed and observed at least every second day, later once a week. The distance between the growing zone of the *S. lacrymans* mycelia and bacterial colony was measured. The production of mycelium-bound and secreted pigments by the fungus were noted. We defined a bacterial strain as inhibitory on fungal growth when the colony size was less than 90% compared to the fungal growth control. Assignment to the category ‘strong inhibiting’ was done when growth was less than 60% compared to the control. Confrontations were performed in triplicates, but in some case not all replicates could be observed for the full 72 days because the plates got contaminated and were removed.

### 2.4. Fluorescence In Situ Hybridization (FISH)

#### 2.4.1. Sampling and Fixation Conditions

*S. lacrymans* tissues (fruiting bodies; Table 1, no. 7, 13, and 15) were washed with sterile distilled water and fixed with 4% Roti^®^-Histofix (Roth, Karlsruhe, Germany) for 1 h. The samples were subsequently dehydrated for 1 h with 50% ethanol at 4 °C and twice with 80% ethanol for 1 h at 4 °C, followed by washing with ethanol 100%. The material was stored at −20 °C until use.

#### 2.4.2. Isolation of the Surface Community by Cuticle Tape Lift

The *S. lacrymans* mycelia and rhizomorphs (Table 1, no. 19 and 20) were used for imprint preparations using double-sided adhesive tape (Tesafilm double sided 7.5 mm × 12 mm, Tesa SE, Norderstedt, Germany) following a modified protocol from Vorholt et al. [56]. Double-sided tape was glued onto microscope slides, without touching the upper side of the tape. The fungal tissue was carefully flattened onto the sticky layer of the tape using an ethanol-wiped, clean glass stick (~1 mm diameter). Tweezers were used to remove the tissue. The tape imprints were fixed with 4% Roti^®^-Histofix (Roth, Karlsruhe, Germany) for 3 h at room temperature. After incubation, the slides were dipped into sterile double-distilled water (ca. 10 s) and subsequently dehydrated for 5 min each in 50%, 80% and 100% ethanol. After drying the slides with compressed air and an additional drying step for 5 min in the dark, the slides were stored at −20 °C.

#### 2.4.3. FISH Probes and Fluorochromes

FITC-, 6FAM-, Cy3-, and Cy5-labelled probes were applied sequentially or simultaneously depending on the required formamide concentrations of the probe (Table 3). The samples stained with multiple probes (EUB338I-IIIw-CY3, BET42aw-6FAM, GAM42aw-6FAM and LGC354ABCw-CY5) were simultaneously analyzed, taking advantage of the non-overlapping emission wavelengths of the fluorochromes (max. excitation/emission in nm: 6FAM 492/518, FITC 490/525, Cy3 548/562 and Cy5 650/670).

#### 2.4.4. (In Tube) FISH

Following a modified protocol from Cardinale et al. [66], small pieces (10–15 mm length) of fixed and rehydrated fruiting body (Table 1, no. 7, 13, and 15) were embedded in OCTTM Tissue-Teks (Sakura, Finetech Europe BV, Zoeterwoude, The Netherlands), rapidly frozen at −25 °C and cut into 30 µm-thick sections with a cryotome. The cryosections were placed in a 1.5 mL microcentrifuge tube and incubated with 1 mg ml^−1^ lysozyme (BioChemika, 105′000 U mg^−1^) for 20 min at room temperature to increase the bacterial cell wall permeability for the FISH probes. Additionally, a 10 mg ml^−1^ concentration of lysozyme was tested. The samples were then rinsed twice with ice-cold 1x PBS (8 g NaCl, 0.2 g KCl, 1.44 g Na_2_HPO_4_2H_2_O, 0.24 g KH_2_PO_4_ and 0.8 l distilled H_2_O). All the hybridizations were performed at 46 °C overnight in a buffer containing 0.9 M NaCl, 0.02 M Tris-HCl (pH 8), 0.01% *v*/*v* sodium dodecyl sulphate (SDS) and 3 ng µL^−1^ of each probe. The concentration of ultrapure formamide (Invitrogen, Waltham, MA, USA) for the FISH probes is given in Table 3. To completely immerse the investigated tissue type sections, 60 µl of hybridization buffer was added. The hybridization buffer was removed the next day and the samples were washed twice with prewarmed (48 °C) washing buffer containing 0.02 M Tris-HCl (pH 8) and 30–450 mM NaCl (Table 3). The washing buffer was adjusted to 5 mM EDTA when the formamide concentration exceeded 20%. The sections were mounted onto a regular microscopic glass slide.

#### 2.4.5. Multiplex FISH

The spatial distribution of bacteria on *S. lacrymans* fruiting bodies was analyzed by sequential hybridization with probes specific for Eubacteria, EUB338 mix, Firmicutes (LGC354ABC mix), and β- and γ-Proteobacteria (BET42aw and GAM42aw) (Table 3). Subsequent hybridizations were started with the probe with the highest formamide concentration, namely, BET42aw and GAM42aw. Next, the probes specific for Firmicutes (LGC354A-C) were applied, and finally the general bacterial probes were used (EUB338II-III). The incubation procedure was the same as above, with the exception that the incubation time at 46 °C was reduced to one hour and the probe hybridization was performed on Teflon-precoated slides (increasement of the hybridization buffer volume to 240 µL). As there were no hints for a particular dispersal pattern in mycelia and rhizomorphs in comparison to the investigated fruiting bodies, these tissues (rhizomorph and mycelium) were excluded from further multiplexing experiments.

#### 2.4.6. FISH for Imprints of the Surface Community on Adhesive Tape

The fixed and frozen double-sided adhesive tape with the imprints of fungal tissue was allowed to warm to room temperature in the dark to prevent precipitation of air humidity. The samples were once more dehydrated in an ethanol series as described above (Section 2.4.2) to remove any residual water. The remaining ethanol was blown off using compressed air, and the slides were allowed to dry in the dark for 10 min. The tape was incubated with 1 mg ml^−1^ lysozyme (BioChemika, 105′000 U mg^−1^) for 20 min at room temperature to increase the bacterial cell wall permeability for the FISH probes. For hybridization with probes specific for Eubacteria (EUB338 mix), the samples were subsequently overlaid with 240 µL hybridization buffer. The incubation procedure was the same as previously described, with the exception that the incubation time at 46 °C was reduced to three hours.

#### 2.4.7. Nucleic Acid and Cell Wall Staining

The FISH-stained sections and the imprints on the double-sided adhesive tape were incubated with 0.7 mg mL^−1^ 4,6-diamino-2-phenyl indole (DAPI) in the dark for 20 min for visualizing the fungal nuclei at room temperature. After washing and drying, the sections were immediately mounted with Vectashield^®^ (BIOZOL-Fit for Science, Eching, Germany) and finally observed under the fully automated Nikon inverted microscope Eclipse Ti2-E. The microscope was equipped with a Zyla sCMOS camera (Oxford Instruments, Abingdon, Great Britain), universal illumination system pE-4000 (CoolLED, Andover, Great Britain), intelligent polarizer Ti2-C-DICP-I (LWD 0.52), control unit TI2-CTRE, piezo nanopositioner combined with a Nano-Drive^®^ controller NIK-C2477 (Mad City Labs Inc., Madison, WI, USA), motorized condenser turret TI2-C-TC-E, pillar for transmitted illumination TI2-D-PD, epi-fluorescence module TI2-LA-FL, a control joystick TI2-S-JS, and a motorized DIC sextuple nosepiece with installed objectives (20×, 40×, and 60×). The maximum projections of an appropriate number of about 0.5 mm-depth optical slices were applied to visualize the fruiting body, mycelia and rhizomorph sections (stacks) using standard settings. The fruiting bodies were cut into 30 µm-thick slices and observed with 20× and 40× magnification, using the following filter sets: F66-413, F36-500, F36-720, F36-740 and F36-760. The digital images were further observed and analyzed with the NIS-Elements Advanced Research software from Nikon (Tokyo, Japan, version 5.11.01). The bacterial cells of 53 individual photos were counted manually after deconvolution (standard settings, type Landweber) using the Nikon software. Hence, we analyzed three fruiting bodies from different locations (Salzburg, Innsbruck 1 and 4; Table 1, no. 15, 7 and 13) in triplicates, or rather quadruplicates, and evaluated a minimum of four pictures of each replicate.

## 3. Results

### 3.1. Microbial Diversity Associated with S. lacrymans Fruiting Bodies, Mycelia and Rhizomorphs

A total of 330 bacterial strains were isolated from 18 fruiting bodies, 13 mycelia, and 10 rhizomorphs samples of *S. lacrymans*. The 16S rRNA gene sequences were generated for 301 of these bacterial isolates (Table S1). After removing sequences shorter than 400 bp, 274 sequences were aligned and their taxonomic affiliation and similarity was analyzed using phylogenetic placement cladograms (Figures S2–S7). The bacterial isolates belonged to 4 phyla, 8 classes, 9 orders, 27 families and 45 genera. Most of the 16S rRNA gene sequences had a high similarity with type species (>98–100%). Most of the isolates (91) grouped with 22 genera of Proteobacteria, 63 isolates with 11 genera of Actinobacteria, and 84 isolates with 7 genera of Firmicutes. The bacterial diversity varied largely among the different developmental stages of *S. lacrymans*, with diversity being higher in the fruiting bodies (27 genera, 125 isolates) and mycelia (26 genera, 65 isolates) compared to the rhizomorph tissue (15 genera, 55 isolates).

**Figure 2:**
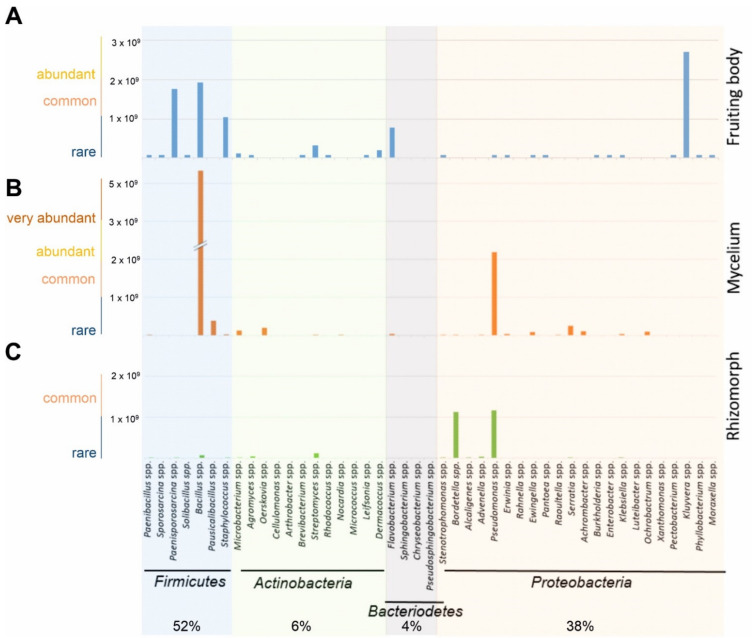
Estimated abundances of bacterial species (in CFU g^−1^) isolated from (**A**) fruiting bodies, (**B**) mycelia and (**C**) rhizomorphs of *S. lacrymans* based on the CFU count derived from an inverse morphotype approach. Due to the reciprocal approach, only relative abundances are indicated. Bacteria were grouped as rare (0–1 × 10^9^ CFU g^−1^), common (1 × 10^9^–2 × 10^9^ CFU g^−1^), abundant (2 × 10^9^–3 × 10^9^ CFU g^−1^), and very abundant (more than 3 × 10^9^ CFU g^−1^). Missing data means that there were either no identifiable colonies, because distinction was not possible due to a highly similar morphotype, or no growth on agar plates.

Approximations from the inverse morphotype approach allowed the estimation that most bacteria from *S. lacrymans* tissues were Gram positive (1.2 × 10^10^ colonies per g fresh weight; 58%). At the phylum level, Firmicutes (52% of all CFU) dominated the bacterial community. The second most abundant group were Proteobacteria (38%). Actinobacteria and Bacteroidetes were found to a lesser extent (6% and 4%, respectively) (Figure 2, Table S2). *Bacillus* sp. were most abundant (37%), followed by *Pseudomonas* sp. (16%) and *Kluyvera* sp. (13%). The bacteria isolated from the fruiting bodies and mycelia were dominated by Firmicutes, while the rhizomorphic population was dominated by Proteobacteria. The rhizomorphs were predominantly colonized by *Pseudomonas* sp. (46%), while *Bacillus* sp. were most abundant (62%) on mycelia, and *Kluyvera* sp. (30%) were the dominant species on fruiting bodies (Figure 2).

**Figure 3:**
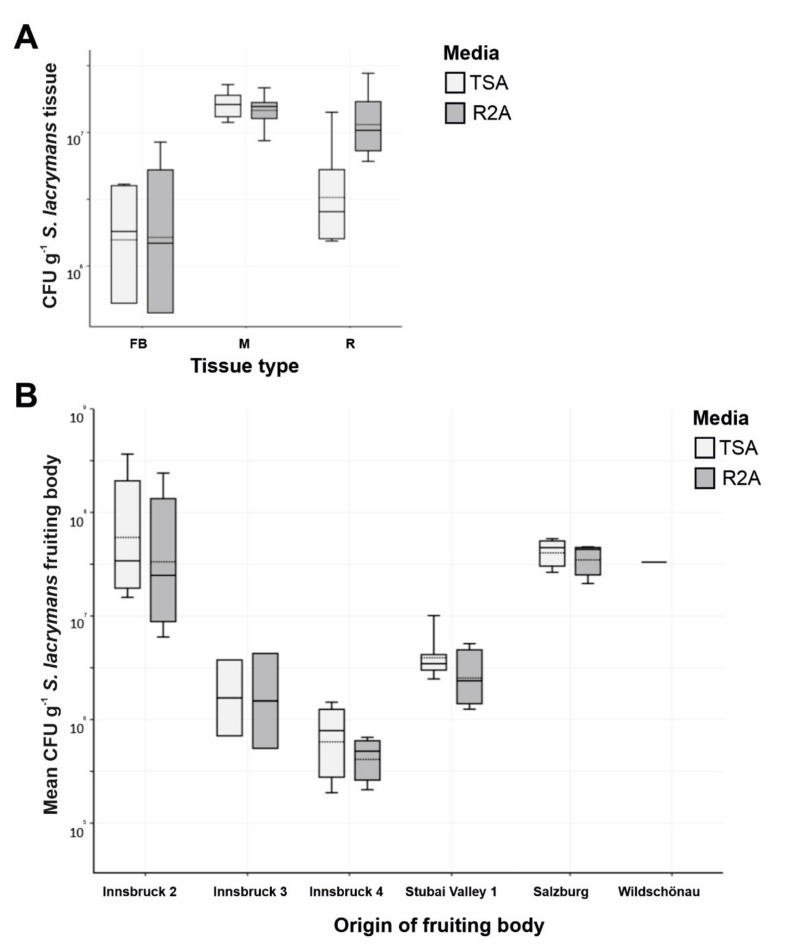
CFU numbers of bacterial colonies on *S. lacrymans* tissue. (**A**) Absolute abundance of bacterial colonies (in CFU g^−1^) derived from different tissues of *S. lacrymans* (Innsbruck 3) (Table 1, No. 12), namely, fruiting bodies (FB), mycelia (M) and rhizomorphs (R); and (**B**) mean CFU g^−1^ of *S. lacrymans* fruiting bodies (Innsbruck 1–4, Stubai valley 1, Salzburg and Wildschönau). Data are displayed in logarithmic scale. Bacteria were cultured on tryptic soy agar (TSA, light grey columns) and R2A agar (R2A, dark columns).

The CFU g^−1^ counts on TSA and R2A agar were comparable. The total number of CFU g^−1^ varied in the different tissue types, with the highest CFU counts derived from mycelium (Figure 3A). On the other hand, the variation was high between sampling sites (Table 1, no. 8, 9, 10, 11, 15 and 18 vs. 12, 13 and 16) (Figure 3B).

### 3.2. Detection and Localization of Bacterial Interaction Partners with Fluorescence In Situ Hybridization (FISH)

**Figure 4:**
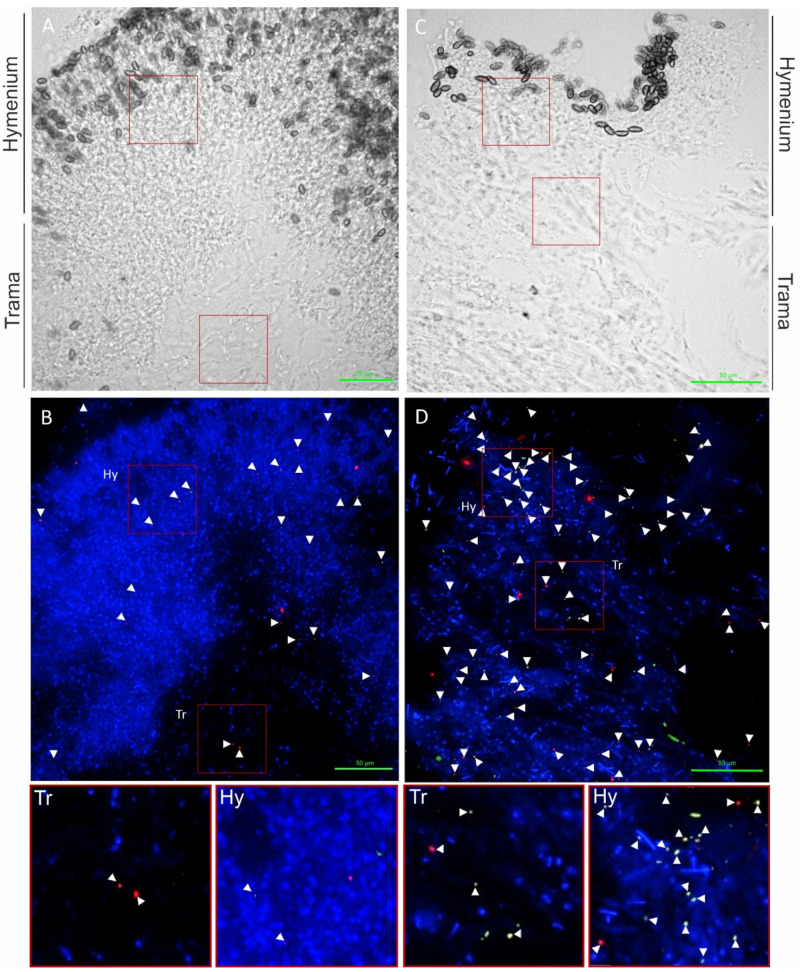
Localization of bacteria on *S. lacrymans* fruiting bodies from Innsbruck 1 (**right**) and Innsbruck 4 (**left**). Firmicutes are displayed as red objects (probe LGC354 Mix-Cy5), β- and γ-Proteobacteria as green objects (BET425aw-FAM and GAM42aw-FAM), and other bacterial cells in orange (EUB338 Mix-Cy3). (**A**) Transmitted light picture; (**B**) overlay of DAPI channel and FISH probe channels; (**C**) transmitted light picture; and (**D**) overlay of DAPI channel and FISH probe channels. Pictures below are enlargements from (**B**,**D**). Signals are highlighted with white arrows. Scale bars are 50 µm.

Spherical bacteria (0.5 to 1.6 µm diameter) could be visualized on the fruiting bodies, mycelium, and rhizomorphic structures of *S. lacrymans*. The bacteria were present mainly as individual cells (Figure 4) and not as biofilms.

**Figure 5:**
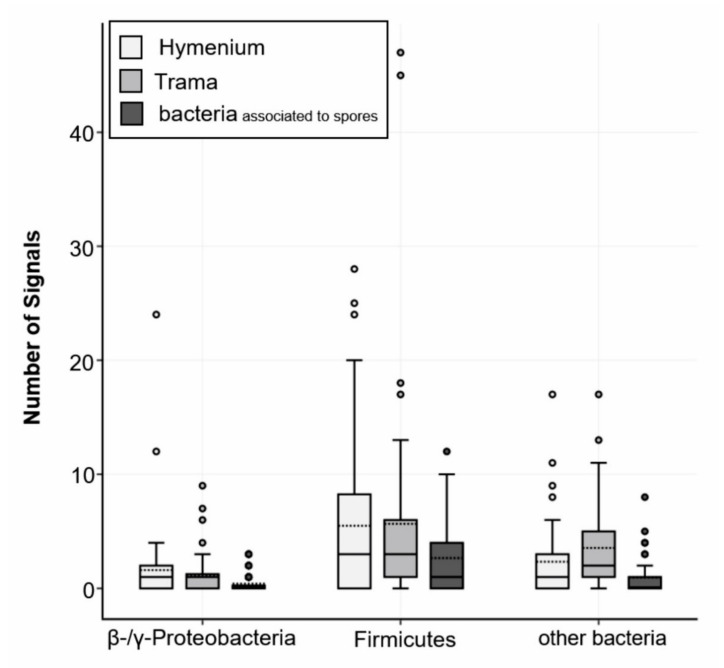
FISH analysis of *S. lacrymans* fruiting bodies. Probes were applied in a multiplexed way. Fungal fruiting bodies from Salzburg, Innsbruck 1 and 4 (Austria; Table 1, no. 7, 13, and 15) were investigated in triplicates. Obtained signals were split into the categories hymenium or trama of fruiting bodies and bacteria associated with spores. Positive signals for β- and γ-Proteobacteria (BET42aw-6FAM and GAM42aw-6FAM probes), Firmicutes (probe LGC354ABCw-CY5) and other bacteria (probe EUB338 Mix-Cy3) are displayed.

Further, 58% of all signals detected with the multiplexed FISH assay were derived from the LGC354ABCw-CY5 probe, indicating that the majority of all visualized bacteria belonged to the phylum Firmicutes. About 13% of all signals were visualized with the BET42aw-6FAM and GAM42aw-6FAM probes (β- and γ-Proteobacteria), and 29% of the signals originated from the general Eubacterial probe (Figure 5). The analysis of all the analyzed slides (multiplex FISH, in total 53 images from three fruiting bodies) gave a mean of 24 signals per section. The medium surface area and thickness of the fungal sections were used to extrapolate and estimate the number of bacteria per cm^3^ fungal structure. Based on the approximation that 1 cm^3^ of fungal fruiting body equals 1 g, 24 signals per section add up to 6.6 × 10^7^ CFU g^−1^ fruiting body. These results are similar to the isolation-based approach, where 10^5^ to 10^8^ CFU g^−1^ fruiting body were identified (Figure 3).

The hymenium contained 45% of bacterial signals, and 34% of bacterial signals were located in the trama of the fruiting bodies. Approximately 17% of all visualized bacterial cells were associated with fungal spores. The fruiting body from Salzburg (Table 1, no. 15) was not entirely mature and therefore had no real hymenium, thus this fruiting body was excluded from these calculations. Notably, all the fruiting bodies contained roughly the same number of bacterial detections (Innsbruck 1–18 signals per image, Innsbruck 4–27 signals per image, and Salzburg 30 signals per image). However, the immature fruiting body (Salzburg) showed a higher content of β- and γ-Proteobacteria (20% vs. 6% and 12% in the mature fruiting bodies). The investigation of mycelial and rhizomorphic tissues with different probes revealed no spatial pattern of bacterial dispersal (Figure S8). Bacteria were not found embedded in a biofilm-like, continuous layer and there was no evidence for intracellular bacteria. The negative controls and nonsense probes (NON-EUB-CY3) were negative. The signals that were larger than 1 µm were generally disregarded (artefacts). In rare instances (less than 1 signal per image) we observed autofluorescence of bacteria (which is particularly known to appear at near infrared. Generally, *S. lacrymans* spores had an autofluorescence, especially at 548 nm (Cy3) and 492 nm (6FAM) excitation.

### 3.3. Enzymatic Activities of Isolated Bacteria

**Figure 6:**
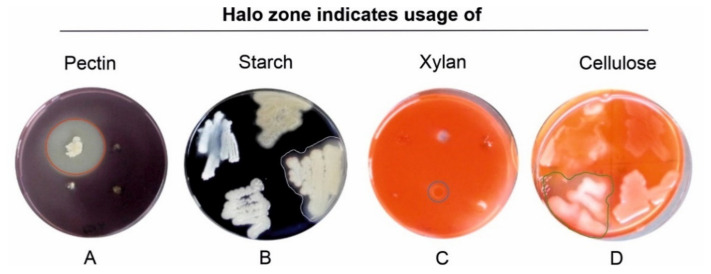
Hydrolysis zones indicate the production of carbohydrate-degrading enzymes. (**A**) Pectinase activity determining PSAM medium (red circle), (**B**) starch degradation determining starch degradation screening agar (SSA) (grey border), (**C**) hemicellulolytic activity determining xylanase screening agar (XSA) (blue circle), and (**D**) cellulolytic activity determining carboxymethylcellulose agar (CMC) (green border). Halos were visualized after flooding the petri dishes with potassium iodide–iodine (**A**,**B**) or 0.1% Congo red solution (**C**,**D**) after incubating for 48–96 h at 25 °C.

The bacteria that were able to depolymerize pectin, xylan, starch or cellulose showed a clear halo zone around their colonies when grown on plates containing the respective biopolymer (Figure 6). In total, 9 of the 327 tested bacteria showed activities of all four enzymes. These were *Serratia* sp. (no. 13), *Paenibacillus* spp. (no. 36 and 79), *Flavobacterium* spp. (No. 41 and 46), one *Arthrobacter* sp. (no. 69), one *Bacillus* sp. (no. 279) and two *Pseudomonas* spp. (91 and 221). It was found that 25% of the isolates were able to degrade pectin, 43% xylan, 17% carboxymethylcellulose, and about 66% were able to depolymerize starch (for detailed information see Table S3 in the supplement); thus, the ability to degrade starch was found most commonly among the bacterial isolates.

### 3.4. Effects of Co-Cultivation on Fungal and Bacterial Growth

**Figure 7:**
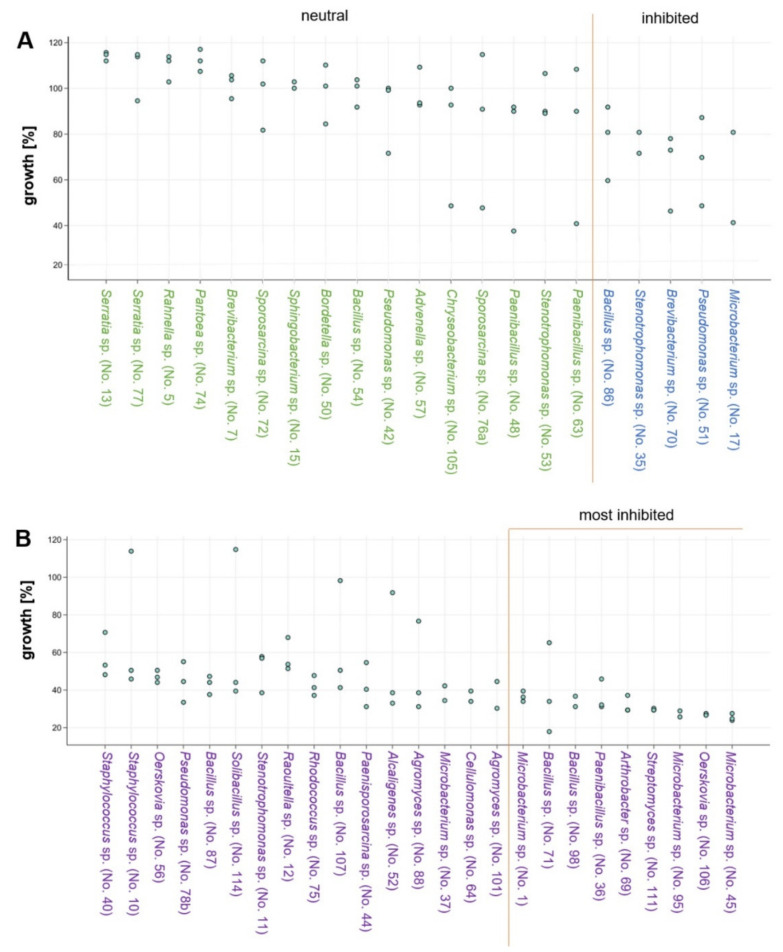
Bacterial co-cultivation with *S. lacrymans*. Fungal growth (in %; in comparison to the growth controls) upon co-incubation with selected bacteria on minimal media. Results from the analysis after 31 days of co-culture are displayed. Two to three replicates per bacterial isolate were performed. Bacteria from left to right are more growth inhibiting. (**A**) Isolates with neutral (90–120% growth; green font) or inhibiting (60–90%; blue font) effects; and (**B**) isolates that were categorized as strongly inhibiting fungal growth (growth less than 60%; purple font).

After 31 days of co-incubation of *S. lacrymans* (no. 1SLIBK2018) with each of the 50 bacterial isolates (Table 2), 16 interactions were neutral or had a slightly positive effect on the mycelial growth of the fungus, with growth rates of 90–117% compared to the mean growth of the control experiment (Figure 7A). Four isolates were moderately growth inhibiting (60–90% growth compared to the control, Figure 7A) while 25 isolates strongly inhibited the mycelial growth of *S. lacrymans* (less than 60% growth in comparison to the control, Figure 7B).

The bacteria that had the highest growth-inhibiting effect on *S. lacrymans* (allowing less than 37% growth) were *Microbacterium* spp. (isolate no. 1, 45, and 95), *Arthrobacter* sp. (isolate 69), *Bacillus* spp. (isolate no. 71 and 98), *Paenibacillus* sp. (isolate 36), *Oerskovia* sp. (isolate 106) and *Streptomyces* sp. (isolate 95) (Figure 7B; last nine isolates). Among the five bacteria strongly inhibiting the growth of *S. lacrymans*, four belonged to *Bacillus* spp. (isolates 71, 87, 98 and 107), which were identified as the dominating group isolated from the *S. lacrymans* structures (Figure 2). However, one *Bacillus* strain (no. 54) was showing neutral behavior. The co-cultivations were again analyzed after 50 days, but no changes compared to the 31 days analyses emerged.

After one week of incubation, mycelium-bound pigments of *S. lacrymans* were visible in all co-cultivation experiments. After 31 days, secreted pigments had emerged in 42 of 49 interactions. No secreted pigment was observed in co-cultivations with isolates belonging to *Brevibacterium* sp. (no. 7), *Sphingobacterium* sp. (no. 15), *Paenibacillus* sp. (no. 48), *Bordetella* sp. (no. 50), *Stenotrophomonas* sp. (no. 53), *Sporosarcina* sp. (no. 72), and *Chryseobacterium* sp. (no. 105).

*S. lacrymans* was also able to inhibit the growth of some bacterial isolates. *Arthrobacter* sp. (no. 59; Figure S9), *Serratia* sp. (no. 77), *Pseudomonas* sp. (no. 78b), and *Cellulomonas* sp. (no. 64) showed reduced growth in the presence of the fungus compared to the control experiment. *Rhodococcus* sp. (no. 75) and *Bacillus* sp. (no. 71) were moderately restricted in growth during the co-cultivation with *S. lacrymans.*

## 4. Discussion

Firmicutes, Proteobacteria, and Actinobacteria dominated the cultivatable bacterial biodiversity associated with *S. lacrymans* in this study. Different developmental stages were associated with different bacterial partners. In general, fungal fruiting bodies can harbor a broad spectrum of microorganisms including bacteria, yeasts, and filamentous fungi [9,27]. Previous studies based on cultivation dependent and independent methods revealed that the bacterial community in decaying wood is dominated by Proteobacteria, but found also bacteria belonging to the phyla Firmicutes, Actinobacteria, and Bacteroidetes [10,12,67]. For instance, the bacterial community coexisting with *Hypholoma fasciculare* in decaying wood were dominated by Proteobacteria, followed by Acidobacteria, Firmicutes and Bacteroidetes [12]. Co-existing bacteria of the mycorrhizal bitter bolete *Tylopilus felleus* were predominantly Proteobacteria, and to a lesser extent Firmicutes, Bacteroidetes, Actinobacteria, and Cyanobacteria [35]. These findings are similar to the results obtained in this study, especially as both approaches, the inverse morphotype assay and FISH microscopy, showed that detectable bacteria were dominated by Firmicutes, while the abundance of Proteobacteria was lower (Figure 2 and Figure 5). Moreover, the CFU g^−1^ fruiting body estimation based on the FISH results was comparable to the CFU counts (Figure 3).

However, it can never be excluded that bacterial co-occurrence is the result of the stochastic ‘mixing’ of *S. lacrymans* with bacteria. For instance, exospore-forming bacteria such as *Streptomyces* sp. are known to be present in moisture-damaged building materials [68,69]. Such random co-occurrences are making it challenging to distinguish true interactions from co-occurrence, especially as bacteria growing on the substrate are isolated as well. Another critical point is the condition of the biological material at the time of sampling. When *S. lacrymans* is diagnosed in buildings, the age and physiology of the fruiting body, mycelia and rhizomorphs vary, the material can be fresh or can already be lysed or desiccated. These variations may also impact microbial communities surrounding the fungus. To minimize the environmental effects on the microbial communities, some samples (Table 1, no. 1 and 4–6 and 16) were excluded from the calculation of CFU g^−1^ fruiting body because those samples were desiccated (data not shown) when collected. Moreover, to counteract the additional putative biases, the remaining material (Table 1, no. 7–15 and 17) was investigated in triplicates to average out drifts. Further, the here used inverse morphotype assay is a qualitative rather than quantitative approximation. The results can be biased by the choice of bacteria isolated from the dilution series plates. This bias was addressed by randomizing the isolates. Many bacterial species have similar morphotypes (e.g., colony shape and color) and cannot be distinguished macroscopically. Cultivation-based approaches are known to miss a large portion of the bacterial biodiversity as most bacteria in environmental samples are uncultivable [70], but assessing the full microbiome via amplicon-based fingerprinting or metagenomic studies was beyond the scope of this study.

Environment-facing structures, like the hymenium of fruiting bodies, were found to be primarily associated with bacteria while the number of bacteria decreased towards the inner parts of the tissues (Figure 4). The analysis with FISH showed similar signal numbers for all three fruiting bodies. The mature fruiting bodies (developing many basidiospores) were associated with β- and γ-Proteobacteria. This could hint at a development-dependent shift in the bacterial composition on fruiting bodies, similar to what has previously been shown for *Cantharellus cibarius* [71], but more targeted research is necessary to answer this question. Our investigation of the mycelial and rhizomorphic tissues of *S. lacrymans* with different single probes revealed individual bacterial cells but no spatial bacterial dispersal pattern (Figure S8). Bacteria were detected on the tissue surface as well as on hyphae and between fungal cells, but not within cells with wide lumen (e.g., rhizomorphs), as is known for instance for *Mortierella elongata*, which harbors the endobacterium *Mycoavidus cysteinexigens* [72]. The analyses of *S. lacrymans* tissue in this study revealed no embedded but individual bacterial cells, in contrast to the reindeer lichen *Cladonia arbuscula*, where bacterial cells were found embedded in a biofilm-like, continuous layer [66].

Nonetheless, one major benefit of isolation-based approaches is that the obtained bacterial isolates are available for further experiments, e.g., to assess their activities and their influence on wood and timber degradation by *S. lacrymans*. As it is long known that bacteria cause structural changes in wood [73], we monitored the enzymatic repertoire of the obtained *S. lacrymans*-derived bacteria. Generally, wood from gymnosperms consists mainly of cellulose (~40%), hemicellulose (~25–30%), and lignin (~25–30%). Additional polymeric substances in wood are pectins and starch [74,75]. Certain bacteria, particularly Actinobacteria (*Rhodococcus*), Firmicutes, Bacteroidetes (*Bacteroides*), α-, β- and γ-Proteobacteria (e.g., *Sphingomonas* and *Burkholderia*), have powerful cellulolytic and pectinolytic enzyme systems, as well as the capability to degrade lignocellulose, while pectinases play a key role in increasing wood permeability [73,76,77]. In this study, 10 out of 327 bacteria produced pectinase, amylase, cellulase, and xylanase including a few isolates of *Serratia* sp., *Paenibacillus* spp., *Flavobacterium* spp., *Arthrobacter* sp., *Bacillus* sp., and *Pseudomonas* spp. The enzymatic power of distinct bacteria, such as *Serratia marcescens,* which is able to hydrolyze carboxymethylcellulose [78], *Bacillus polymyxa* that can hydrolyze pectin and holocellulose [79], and *Pseudomonas* sp. that are known for their phenol and aromatics degrading ability [80], is well documented. In addition, *Burkholderia* sp. are known as efficient mineral-weathering and nitrogen-fixing bacteria [81]. It therefore might be worth further characterizing the microbial collection established in this study for the ability to fix nitrogen, as especially nitrogen is scarce in wood, and fungi can overcome this deficiency by translocating the element from the surrounding environment where it is accumulated by N-fixing bacteria [82]. Another interesting point would be to test the isolated bacteria for chitinolytic enzymes as this might give hints towards their ability to feed on fungal tissue. Additionally, increased leakage of sugars from the fungal wood decay could be synergistic for bacterial growth or could change the bacterial diversity (i.e., slow growth in the beginning, and faster growth in proceeded decay stages), but this needs more research. The streaking-out design for the enzymatic activity assay is useful to assess whether there is or is not enzymatic activity, but we cannot make any quantitative comparisons between bacterial isolates. As we put several strains on the same plate, we also cannot rule out that certain bacteria could have inhibited the activities of others through volatiles or soluble compounds.

Co-cultivations are frequently used to trigger the production of bioactive metabolites by microorganisms, as axenic cultures do not reflect the natural situation [83]. On the one hand bacteria may be antagonistic to fungi and competing for resources [20], on the other hand they can be beneficial as certain bacterial metabolic products may function as growth factors for fungi [5]. We found that the bacteria with the highest growth-inhibiting effect on *S. lacrymans* were *Microbacterium* spp., *Arthrobacter* sp., *Bacillus* spp., *Paenibacillus* sp., *Oerskovia* sp. and *Streptomyces* sp. (Figure 7B; last nine isolates). The ability of all of these mentioned bacteria to use (fungal) chitin as a carbon source is well documented [84,85,86,87,88], and *B. amyloliquefaciens* has as well been found to antagonize the filamentous fungi *Botrytis cinerea* and *Fusarium solani* [89]. Otherwise, *S. lacrymans* could inhibit the growth of some bacterial isolates too, although it was shown that *S. lacrymans* has poor antagonistic behavior against other fungi [90]. We saw that genera, which cause growth impairment of *S. lacrymans,* and bacterial genera, which are decreased in their growth by the fungus, overlapped. This indicates that a cross-talk took place which resulted in the enhanced production of bioactive metabolites by the interaction partners. This over-production could impair the growth of the bacterial counterpart, and vice versa, the bacterium might start to produce substances that inhibit the fungus.

The obtained results showed heterogeneity among the replicates because of the uneven growth rates of the *S. lacrymans* strains used, which could not be prevented despite very stringent methodological standardizations (standardized media preparation, light/dark conditions, and preparation of fungal and bacterial pre-cultures). To address these growth differences relative growth values were compared rather than absolute values, resulting in comparable patterns across the experiments. Co-cultivation pairings with the strongest fungal growth inhibition showed similar results over all the replicates; hence, the real strong antagonistic behavior of bacteria is not masked by biological variation. There were hints that the bacterial strains causing strong growth impairment of *S. lacrymans* also showed good performance in the enzyme activity test. This may suggest that a broad repertoire of enzymatic features is useful in the warfare for habitat and nutrients. The fungal efforts in this warfare are for instance well noticeable by the selective effects of white-rot basidiomycetes on the bacterial community during wood decay [20]. Therefore, the competition between bacteria and fungi may influence the establishment and subsequent changes in the bacterial community.

## 5. Conclusions

This study aimed at a first characterization of the mechanistic details of *S. lacrymans* bacteria interaction, and therefore cultures of the bacterial isolates were favored over mere DNA analysis, which simply gives information on the presence/absence of species. Importantly, both approaches, the culture-dependent assay and FISH microscopy, while based on different principles, gave similar results, and consequently, the outcome gains more meaningfulness. The 16S rRNA gene was used for the phylogenetic placement of the bacterial microbiota of *S. lacrymans*. Nonetheless, future studies that include uncultured bacteria identified via metagenomic approaches are needed, as well as studies on the fungal interaction partners of *S. lacrymans*. The exact mechanisms of the interaction between *S. lacrymans* and its bacterial microbiota remain also open. In summary, we provide the first insights into the bacterial interaction partners of the dry rot fungus *S. lacrymans*, among which we found a dominance of Gram-positive bacteria. This could be emphasized by FISH experiments and the better signals from the Firmicutes-specific probe. The bacterial isolates were tested for their enzymatic repertoire, especially for enzymes, which are relevant in the dead wood environment. The results of these experiments hint at the enormous power of bacteria in producing different enzyme types. Especially the single isolates of *Microbacterium* sp., *Flavobacterium* sp., *Arthrobacter* sp., and *Bacillus* sp. were, amongst others, promising candidates for further investigating their enzymatic repertoire and, based on their antagonistic activity, their secondary metabolites. Whether these bacteria have as well an effect on the wood-decaying properties of *S. lacrymans* remains to be revealed. This study provides new and relevant insights into the *S. lacrymans* bacterial microbiota and paves the way for future studies on the recruitment, function and evolution of fungi-associated bacteria.

## Figures and Tables

**Table 1:** *Serpula lacrymans* collections used in this study.

No.	Origin	Fruiting Body	Mycelia	Rhizomorph	Sampling Date	Bacterial Isolation	CFU Assay	FISH
1	Außerfern	2	1	1	13 December 2018	X		
2	Brixen valley 1	0	2	0	19 October 2018	X		
3	Brixen valley 2	0	1	0	2 May 2019	X		
4	Brixen valley 2	1	1	0	19 June 2019	X		
5	Buchkirchen	1	0	1	14 June 2019	X		
6	Grieskirchen	1	1	1	13 June 2019	X		
7	Innsbruck 1	2	3	2	7 November 2018	X	X	X
8	Innsbruck 2	2	0	0	22 March 2019	X	X	
9	Innsbruck 2	1	0	0	2 July 2019	X	X	
10	Innsbruck 2	1	1	1	16 August 2019	X	X	
11	Innsbruck 2	1	0	1	6 September 2019	X	X	
12	Innsbruck 3	1	1	1	30 October 2019	X	X	
13	Innsbruck 4	1	0	0	18 November 2019	X	X	X
14	Pitz valley	1	1	1	16 May 2019	X	X	
15	Salzburg	1	0	0	31 October 2019	X	X	X
16	Stubai valley 1	1	0	0	14 October 2019	X		
17	Stubai valley 2	0	1	1	28 November 2019	X	X	
18	Wildschönau	1	0	0	27 June 2018	X		
19	Traunkirchen	0	0	1	26 June 2020			X
20	Ziller valley	0	1	0	2 July 2020			X

X: analyses made with the sample

**Table 2:** Bacterial isolates used for co-culturing experiments.

Isolate No.	Taxonomy	Isolate No.	Taxonomy
1	*Microbacterium* sp.	41	*Flavobacterium* sp.
17	*Microbacterium* sp.	46	*Flavobacterium* sp.
37	*Microbacterium* sp.	50	*Bordetella* sp.
45	*Microbacterium* sp.	52	*Alcaligenes* sp.
60	*Microbacterium* sp.	54	*Bacillus* sp.
95	*Microbacterium* sp.	71	*Bacillus* sp.
99	*Microbacterium* sp.	86	*Bacillus* sp.
5	*Rahnella* sp.	87	*Bacillus* sp.
7	*Brevibacterium* sp.	92	*Bacillus* sp.
70	*Brevibacterium* sp.	98	*Bacillus* sp.
10	*Staphylococcus* sp.	107	*Bacillus* sp.
40	*Staphylococcus* sp.	56	*Oerskovia* sp.
11	*Stenotrophomonas* sp.	106	*Oerskovia* sp.
35	*Stenotrophomonas* sp.	57	*Advenella* sp.
53	*Stenotrophomonas* sp.	64	*Cellulomonas* sp.
12	*Raoultella* sp.	69	*Arthrobacter* sp.
13	*Serratia* sp.	72	*Sporosarcina* sp.
77	*Serratia* sp.	74	*Pantoea* sp.
15	*Sphingobacterium* sp.	75	*Rhodococcus* sp.
36	*Paenibacillus* sp.	76a)	*Sporosarcina* sp.
48	*Paenibacillus* sp.	81	*Erwinia* sp.
63	*Paenibacillus* sp.	88	*Agromyces* sp.
42	*Pseudomonas* sp.	101	*Agromyces* sp.
51	*Pseudomonas* sp.	105	*Chryseobacterium* sp.
78b)	*Pseudomonas* sp.	111	*Streptomyces* sp.
44	*Paenisporosarcina* sp.	114	*Solibacillus* sp.

No.: Number.

**Table 3:** FISH probes used.

Name	Sequence (5′-3′)	Target	Formamide Conc. [%] ^a^	Fluorophore	NaCl Con. Washing Buffer [mM]	Reference
EUB338w ^b^	gctgcctcccgtaggagt	Most bacteria	10	Cy3/Cy5	450	[57]
EUB338IIw ^b^	gcagccacccgtaggtgt	*Planctomycetales*	10	Cy3/Cy5	450	[58]
EUB338IIIw ^b^	gctgccacccgtaggtgt	*Verrucomicrobiales*	10	Cy3/Cy5	450	[58]
ALF968 ^b^	ggtaaggttctgcgcgtt	Alphaproteobacteria, except *Rickettsiales*	40	Cy3	56	[59]
BET42aw ^b^	gccttcccacttcgttt	Betaproteobacteria	40	6FAM	56	[60]
GAM42aw ^b^	gccttcccacatcgttt	Gammaproteobacteria	40	6FAM	56	[60]
LGC354Aw ^b^	tggaagattccctactgc	Firmicutes (low G + C Gram-positive bacteria)	35	Cy5	80	[61]
LGC354Bw ^b^	cggaagattccctactgc	Firmicutes (low G + C Gram-positive bacteria)	35	Cy5	80	[61]
LGC354Cw ^b^	ccgaagattccctactgc	Firmicutes (low G + C Gram-positive bacteria)	35	Cy5	80	[61]
HGC69A	tatagttaccaccgccgt	Actinobacteria (high G + C Gram-positive bacteria)	25	Cy5	160	[62]
R-FL615	cactgcaatcgttgagcga	Bacteroidetes	35	Cy5	80	[63]
PSE1284	gatccggactacgatcggttt	*Pseudomonadales*	30	Cy5	220	[64]
NONEUB	actcctacgggaggcagc		^c^	Cy3	^c^	[65]

^a^ The indicated concentrations of formamide are intended for hybridizations at 46 °C. ^b^ Used together in equimolar concentration. ^c^ Used as a negative control at the same formamide/NaCl concentration for the positive FISH probe.

## Data Availability

The data presented in this study are available within the article and the Supplementary Materials.

## Supplementary Materials

The following are available online at https://www.mdpi.com/article/10.3390/jof7050354/s1; Figure S1: isolation and characterization of bacteria from tissues of *Serpula lacrymans*—including 16S rRNA gene fingerprint and inverse morphotype assay; Figure S2: cladogram of bacteria belonging to the phylum Bacteroidetes; Figure S3: cladogram of bacteria belonging to the phylum Proteobacteria (part A); Figure S4: cladogram of bacteria belonging to the phylum Proteobacteria (part B); Figure S5: cladogram of bacteria belonging to the phylum Firmicutes (part A); Figure S6: cladogram of bacteria belonging to the phylum Firmicutes (part B); figure S7: cladogram of bacteria belonging to the phylum Actinobacteria; Figure S8: localization of bacteria on *S. lacrymans* rhizomorphs and mycelium; Figure S9: inhibition of bacterial growth by *S. lacrymans*; Table S1: accession numbers for sequences of bacterial isolates retrieved from NCBI; Table S2: estimated abundances of bacterial species derived from different tissues of *S. lacrymans*; and Table S3: results of pectinase, xylanase, cellulase and amylase production assays.
