# Supplementary material for: Microbiota Associated with Different Developmental Stages of the Dry Rot Fungus Serpula lacrymans
Source: J Fungi (Basel). 2021 Apr 30;7(5):354. doi: 10.3390/jof7050354 (PMC8147175; doi:10.3390/jof7050354)
Supplement: Supplementary file 1 [file jof-07-00354-s001.zip › jof-1184281-supplementary.pdf]

Supplementary materials

Microbiota associated with different developmental stages of the dry rot fungus *Serpula lacrymans*

Julia Embacher[1], Sigrid Neuhauser[1], Susanne Zeilinger[1], and Martin Kirchmair[1*]

[1]Department of Microbiology, University of Innsbruck, Technikerstrasse 25, 6020 Innsbruck, Austria

*Correspondence: martin.kirchmair@uibk.ac.at

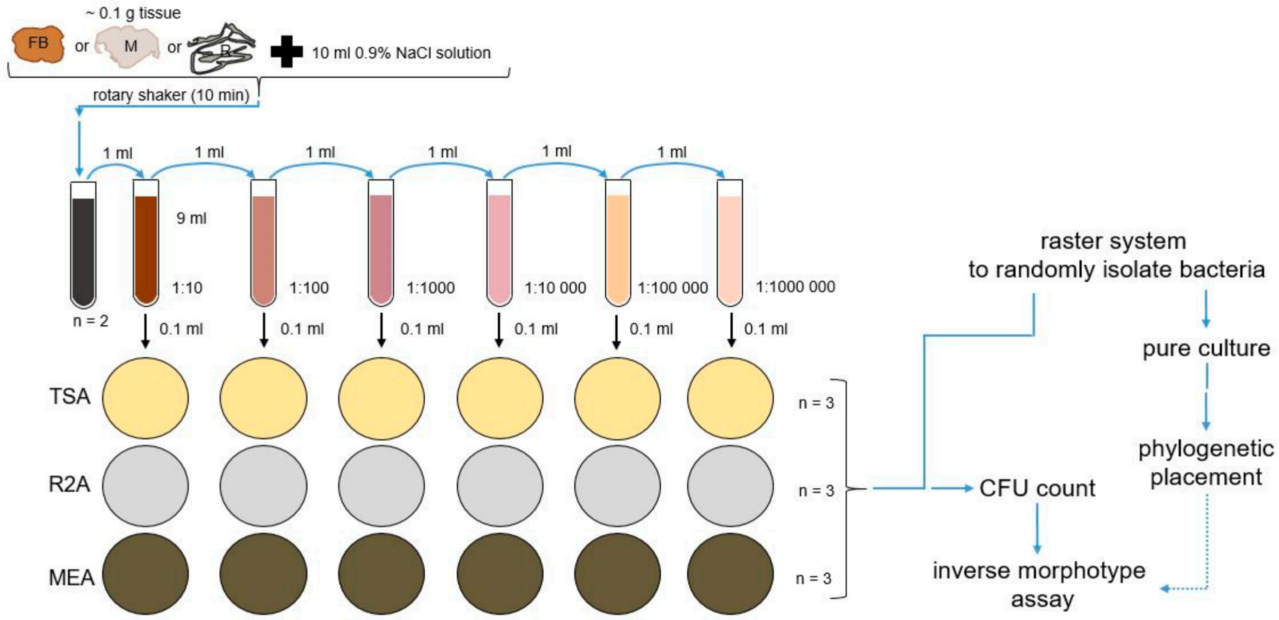

Figure S1 | Isolation and characterization of bacteria from tissues of *Serpula lacrymans* – including 16S rRNA gene fingerprint and inverse morphotype assay: 0.1 - 1 g fungal tissue (FB: fruiting body, M: mycelia, R: rhizomorphs) were homogenized in 10 ml 0.9% NaCl solution. 100 µL of this suspension (or of a serial dilution therefrom) were plated on tryptone soy agar (TSA), R2A agar, and malt extract agar (MEA) in triplicates and incubated for 7 days at 25 °C. Petri dishes were checked daily. Petri dishes with 30 to 100 CFU were used for counting. Bacterial colonies were divided into morphotypes and the CFU of each morphotype were counted. From each plate 6 CFU were selected randomly (raster system) and bacterial pure cultures were created and identified upon their 16S rRNA gene. Each morphotype could therefore be assigned to 16S rRNA gene sequences.

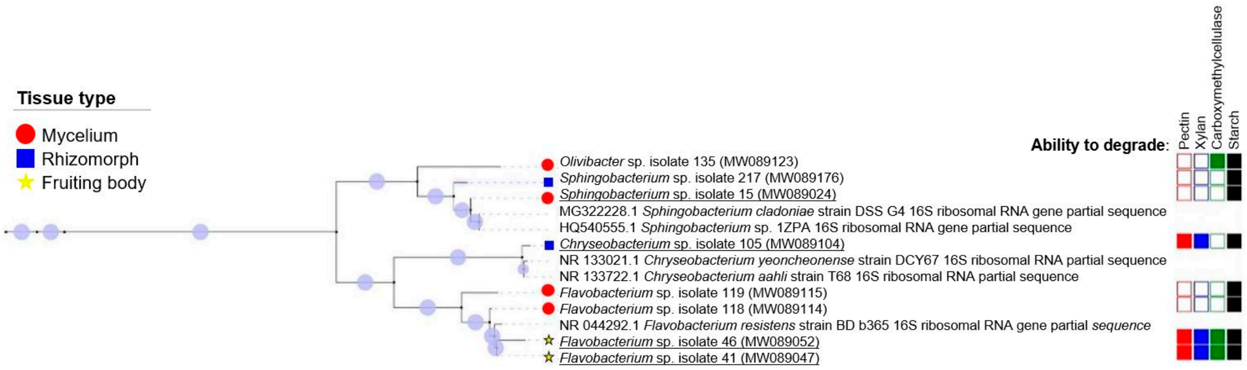

Figure S2 | Cladogram of bacteria belonging to the phylum Bacteroidetes (PHYML). 16S rRNA gene sequences show the relationships between bacteria isolated from *S. lacrymans* tissue types. Most 16S rRNA gene sequences had high similarity with type species (>98%-100%); final assignment at genera level was based on the best blast result. 13 sequences were used for tree creation. Light blue circles indicate a chi^2 statistics greater than 0.75. Reference sequences were retrieved from NCBI. Symbols that indicate tissue of isolation: red circle = bacteria isolated from mycelium; star = bacteria isolated from fruiting body and blue rectangle = bacteria isolated from rhizomorphs. Symbols on the right: red rectangle indicates ability to degrade pectin, blue rectangle indicates ability to degrade xylan, green rectangle indicates ability to degrade carboxymethylcellulose and black rectangle indicates ability to degrade starch. Underlined isolates were tested in co-culture assays.

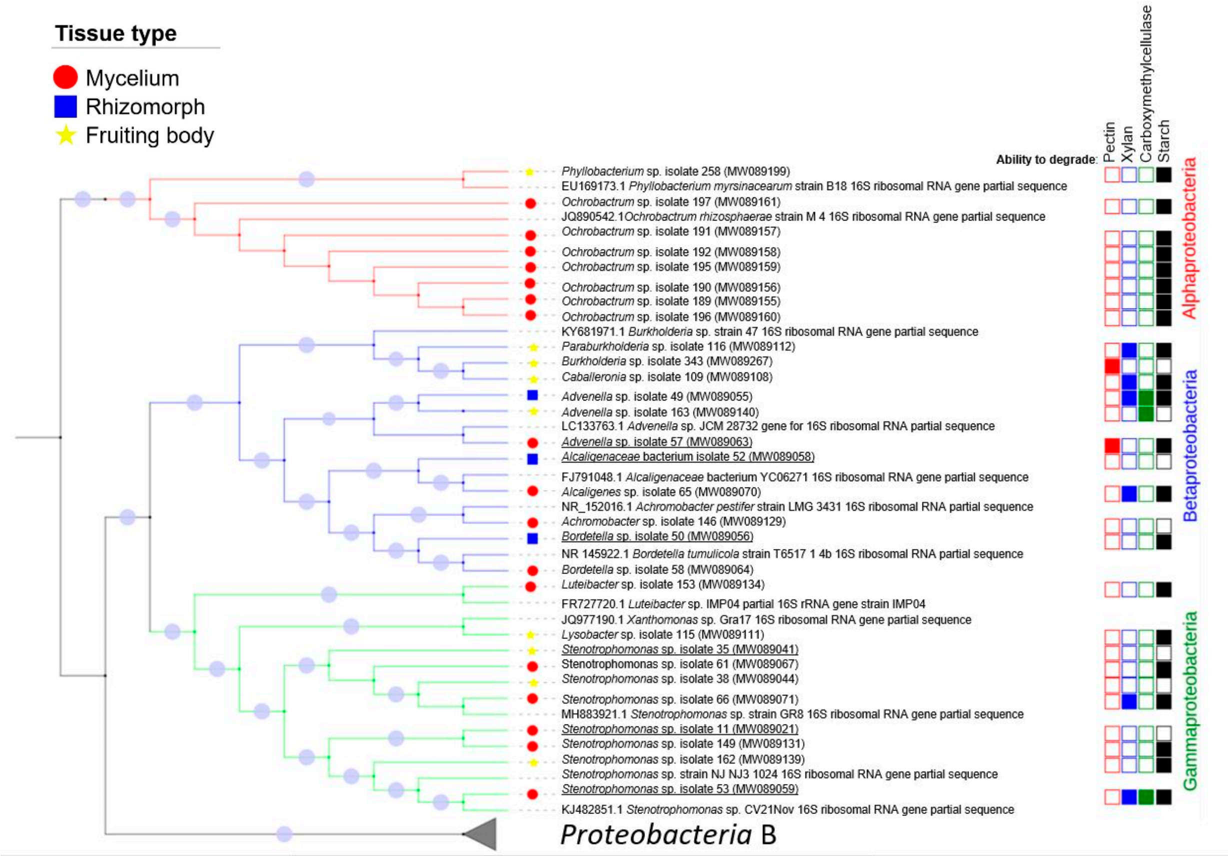

Figure S3 | Cladogram of bacteria belonging to the phylum Proteobacteria (part A) (PHYML). 16S rRNA gene sequences show the relationships between bacteria isolated from *S. lacrymans* tissue types. Most 16S rRNA gene sequences had high similarity with type species (>98%-100%); final assignment at genera level was based on the best blast result. 41 sequences were used for tree creation. Light blue circles indicate a chi^2 statistics greater than 0.75. Reference sequences were retrieved from NCBI. Symbols that indicate tissue of isolation: red circle = bacteria isolated from mycelium; star = bacteria isolated from fruiting body and blue rectangle = bacteria isolated from rhizomorphs. Symbols on the right: red rectangle indicates ability to degrade pectin, blue rectangle indicates ability to degrade xylan, green rectangle indicates ability to degrade carboxymethylcellulose and black rectangle indicates ability to degrade starch. Red branches are Alphaproteobacteria, blue branches are Betaproteobacteria and green branches are Gammaprotobacteria. For *Proteobacteria* part B see figure S4. Underlined isolates were tested in co-culture assays

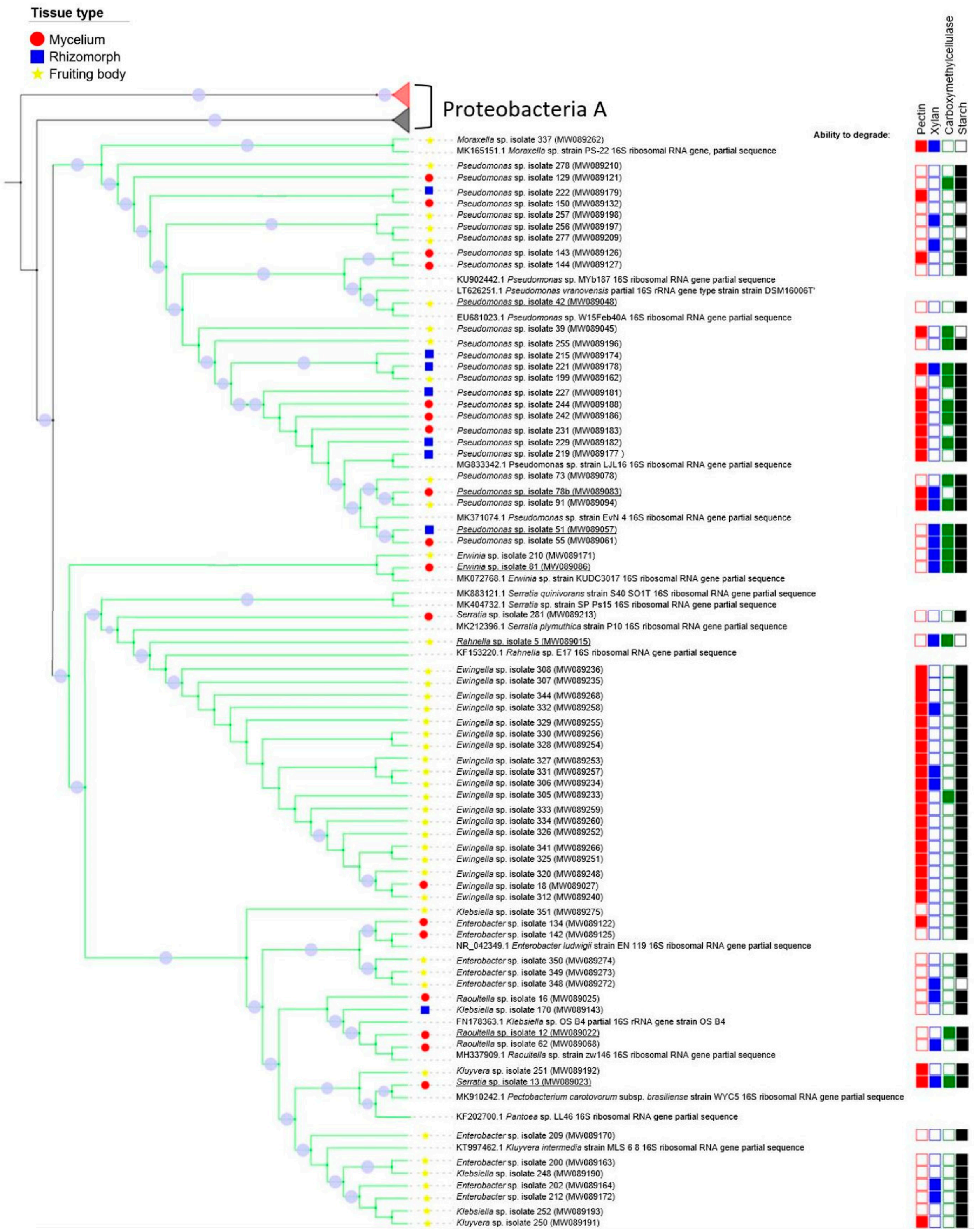
Tissue type
Mycelium
Rhizomorph
Fruiting body
Proteobacteria A
Ability to degrade:
Pectin
Xylan
Carboxymethylcellulase
Starch
Moraxella sp. isolate 337 (MW089262)
MK165151.1 Moraxella sp. strain PS-22 16S ribosomal RNA gene, partial sequence
Pseudomonas sp. isolate 278 (MW089210)
Pseudomonas sp. isolate 129 (MW089121)
Pseudomonas sp. isolate 222 (MW089179)
Pseudomonas sp. isolate 150 (MW089132)
Pseudomonas sp. isolate 257 (MW089198)
Pseudomonas sp. isolate 256 (MW089197)
Pseudomonas sp. isolate 277 (MW089209)
Pseudomonas sp. isolate 143 (MW089126)
Pseudomonas sp. isolate 144 (MW089127)
KU902442.1 Pseudomonas sp. MYb187 16S ribosomal RNA gene partial sequence
LT626251.1 Pseudomonas vranovensis partial 16S rRNA gene type strain strain DSM16006T'
Pseudomonas sp. isolate 42 (MW089048)
EU681023.1 Pseudomonas sp. W15Feb40A 16S ribosomal RNA gene partial sequence
Pseudomonas sp. isolate 39 (MW089045)
Pseudomonas sp. isolate 255 (MW089196)
Pseudomonas sp. isolate 215 (MW089174)
Pseudomonas sp. isolate 221 (MW089178)
Pseudomonas sp. isolate 199 (MW089162)
Pseudomonas sp. isolate 227 (MW089181)
Pseudomonas sp. isolate 244 (MW089188)
Pseudomonas sp. isolate 242 (MW089186)
Pseudomonas sp. isolate 231 (MW089183)
Pseudomonas sp. isolate 229 (MW089182)
Pseudomonas sp. isolate 219 (MW089177)
MG833342.1 Pseudomonas sp. strain LJL16 16S ribosomal RNA gene partial sequence
Pseudomonas sp. isolate 73 (MW089078)
Pseudomonas sp. isolate 78b (MW089083)
Pseudomonas sp. isolate 91 (MW089094)
MK371074.1 Pseudomonas sp. strain EvN 4 16S ribosomal RNA gene partial sequence
Pseudomonas sp. isolate 51 (MW089057)
Pseudomonas sp. isolate 55 (MW089061)
Erwinia sp. isolate 210 (MW089171)
Erwinia sp. isolate 81 (MW089086)
MK072768.1 Erwinia sp. strain KUDC3017 16S ribosomal RNA gene partial sequence
MK883121.1 Serratia quinivorans strain S40 SO1T 16S ribosomal RNA gene partial sequence
MK404732.1 Serratia sp. strain SP Ps15 16S ribosomal RNA gene partial sequence
Serratia sp. isolate 281 (MW089213)
MK212396.1 Serratia plymuthica strain P10 16S ribosomal RNA gene partial sequence
Rahnella sp. isolate 5 (MW089015)
KF153220.1 Rahnella sp. E17 16S ribosomal RNA gene partial sequence
Ewingella sp. isolate 308 (MW089236)
Ewingella sp. isolate 307 (MW089235)
Ewingella sp. isolate 344 (MW089268)
Ewingella sp. isolate 332 (MW089258)
Ewingella sp. isolate 329 (MW089255)
Ewingella sp. isolate 330 (MW089256)
Ewingella sp. isolate 328 (MW089254)
Ewingella sp. isolate 327 (MW089253)
Ewingella sp. isolate 331 (MW089257)
Ewingella sp. isolate 306 (MW089234)
Ewingella sp. isolate 305 (MW089233)
Ewingella sp. isolate 333 (MW089259)
Ewingella sp. isolate 334 (MW089260)
Ewingella sp. isolate 326 (MW089252)
Ewingella sp. isolate 341 (MW089266)
Ewingella sp. isolate 325 (MW089251)
Ewingella sp. isolate 320 (MW089248)
Ewingella sp. isolate 18 (MW089027)
Ewingella sp. isolate 312 (MW089240)
Klebsiella sp. isolate 351 (MW089275)
Enterobacter sp. isolate 134 (MW089122)
Enterobacter sp. isolate 142 (MW089125)
NR_042349.1 Enterobacter ludwigii strain EN 119 16S ribosomal RNA gene partial sequence
Enterobacter sp. isolate 350 (MW089274)
Enterobacter sp. isolate 349 (MW089273)
Enterobacter sp. isolate 348 (MW089272)
Raoultella sp. isolate 16 (MW089025)
Klebsiella sp. isolate 170 (MW089143)
FN178363.1 Klebsiella sp. OS B4 partial 16S rRNA gene strain OS B4
Raoultella sp. isolate 12 (MW089022)
Raoultella sp. isolate 62 (MW089068)
MH337909.1 Raoultella sp. strain zw146 16S ribosomal RNA gene partial sequence
Kluyvera sp. isolate 251 (MW089192)
Serratia sp. isolate 13 (MW089023)
MK910242.1 Pectobacterium carotovorum subsp. brasiliense strain WYC5 16S ribosomal RNA gene partial sequence
KF202700.1 Pantoea sp. LL46 16S ribosomal RNA gene partial sequence
Enterobacter sp. isolate 209 (MW089170)
KT997462.1 Kluyvera intermedia strain MLS 6 8 16S ribosomal RNA gene partial sequence
Enterobacter sp. isolate 200 (MW089163)
Klebsiella sp. isolate 248 (MW089190)
Enterobacter sp. isolate 202 (MW089164)
Enterobacter sp. isolate 212 (MW089172)
Klebsiella sp. isolate 252 (MW089193)
Kluyvera sp. isolate 250 (MW089191)

Figure S4 | Cladogram of bacteria belonging to the phylum Proteobacteria (part B) (PHYML). 16S rRNA gene sequences show the relationships between bacteria isolated from *S. lacrymans* tissue types. Most 16S rRNA gene sequences had high similarity with type species (>98%-100%); final assignment at genera level was based on the best blast result. 138 sequences were used for tree creation. Light blue circles indicate a chi^2 statistics greater than 0.75. Reference sequences were retrieved from NCBI. Symbols that indicate tissue of isolation: red circle = bacteria isolated from mycelium; star = bacteria isolated from fruiting body and blue rectangle = bacteria isolated from rhizomorphs. Symbols on the right: red rectangle indicates ability to degrade pectin, blue rectangle indicates ability to degrade xylan, green rectangle indicates ability to degrade carboxymethylcellulose and black rectangle indicates ability to degrade starch. Green branches show Gammaproteobacteria. For Proteobacteria part A see figure S3. Underlined isolates were tested in co-culture assays.

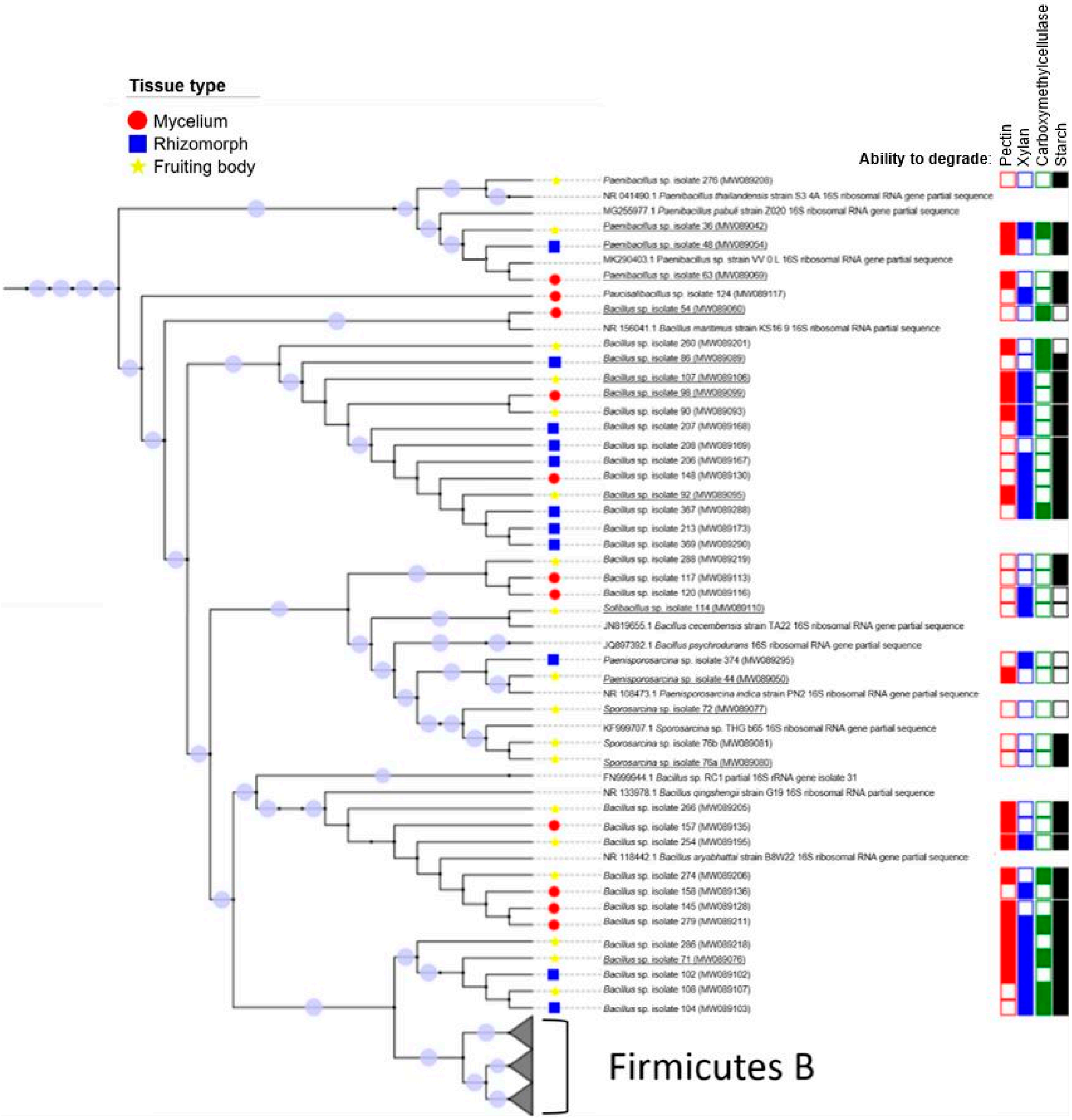

Figure S5 | Cladogram of bacteria belonging to the phylum Firmicutes (part A) (PHYML). 16S rRNA gene sequences show the relationships between bacteria isolated from *S. lacrymans* tissue types. Most 16S rRNA gene sequences had high similarity with type species (>98%-100%); final assignment at genera level was based on the best blast result. 102 sequences were used for tree creation. Light blue circles indicate a chi^2 statistics greater than 0.75. Reference sequences were retrieved from NCBI. Symbols that indicate tissue of isolation: red circle = bacteria isolated from mycelium; star = bacteria isolated from fruiting body and blue rectangle = bacteria isolated from rhizomorphs. Symbols on the right: red

rectangle indicates ability to degrade pectin, blue rectangle indicates ability to degrade xylan, green rectangle indicates ability to degrade carboxymethylcellulose and black rectangle indicates ability to degrade starch. For Firmicutes part B see figure S6. Underlined isolates were tested in co-culture assays.

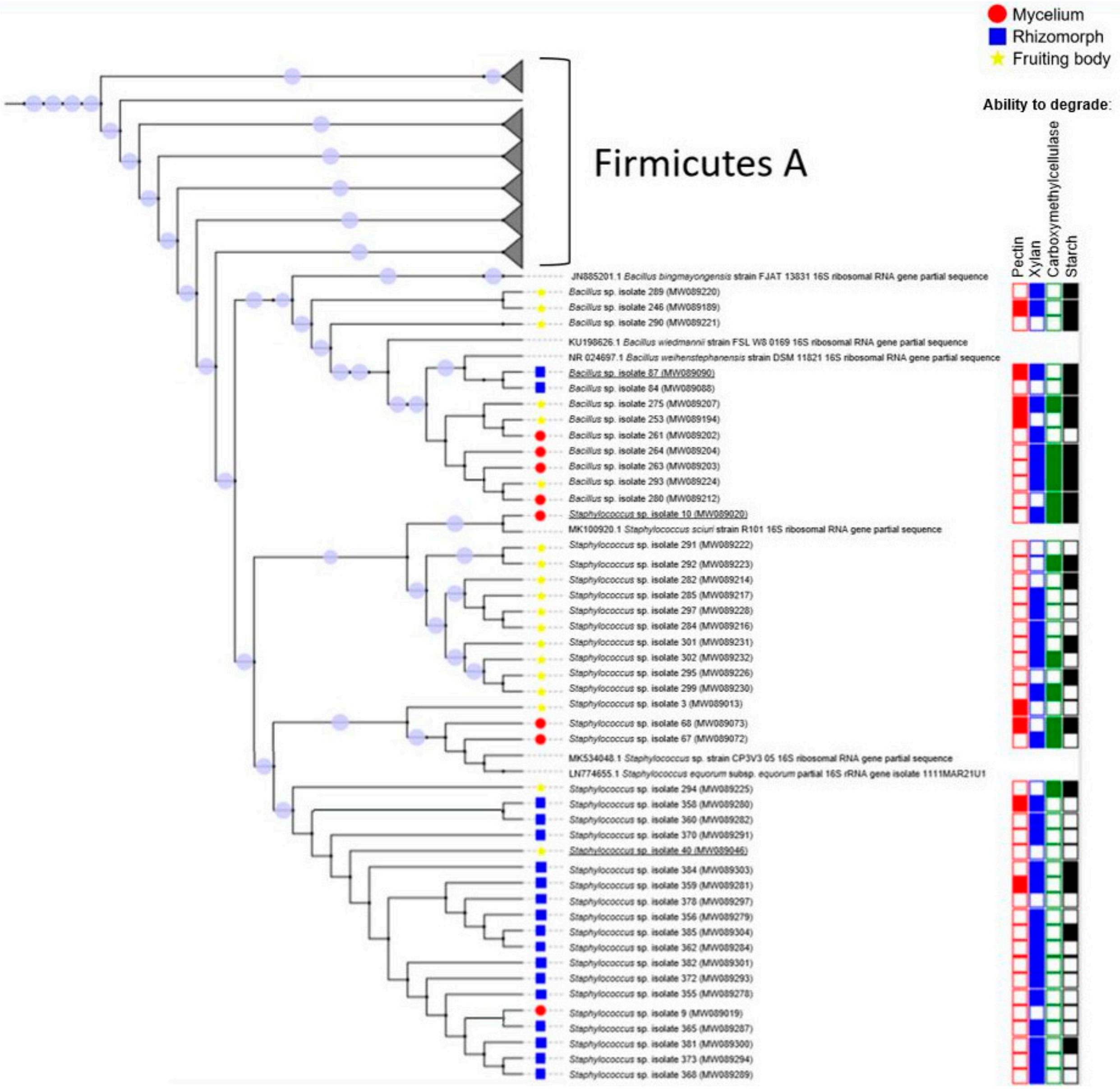

Figure S6 | Cladogram of bacteria belonging to the phylum Firmicutes (part B) (PHYML). 16S rRNA gene sequences show the relationships between bacteria isolated from *S. lacrymans* tissue types. Most 16S rRNA gene sequences had high similarity with type species (>98%-100%); final assignment at genera level was based on the best blast result. 102 sequences were used for tree creation. Light blue circles indicate a chi^2 statistics greater than

0.75. Reference sequences were retrieved from NCBI. Symbols that indicate tissue of isolation: red circle = bacteria isolated from mycelium; star = bacteria isolated from fruiting body and blue rectangle = bacteria isolated from rhizomorphs. Symbols on the right: red rectangle indicates ability to degrade pectin, blue rectangle indicates ability to degrade xylan, green rectangle indicates ability to degrade carboxymethylcellulose and black rectangle indicates ability to degrade starch. For Firmicutes part A see figure S5. Underlined isolates were tested in co-culture assays.

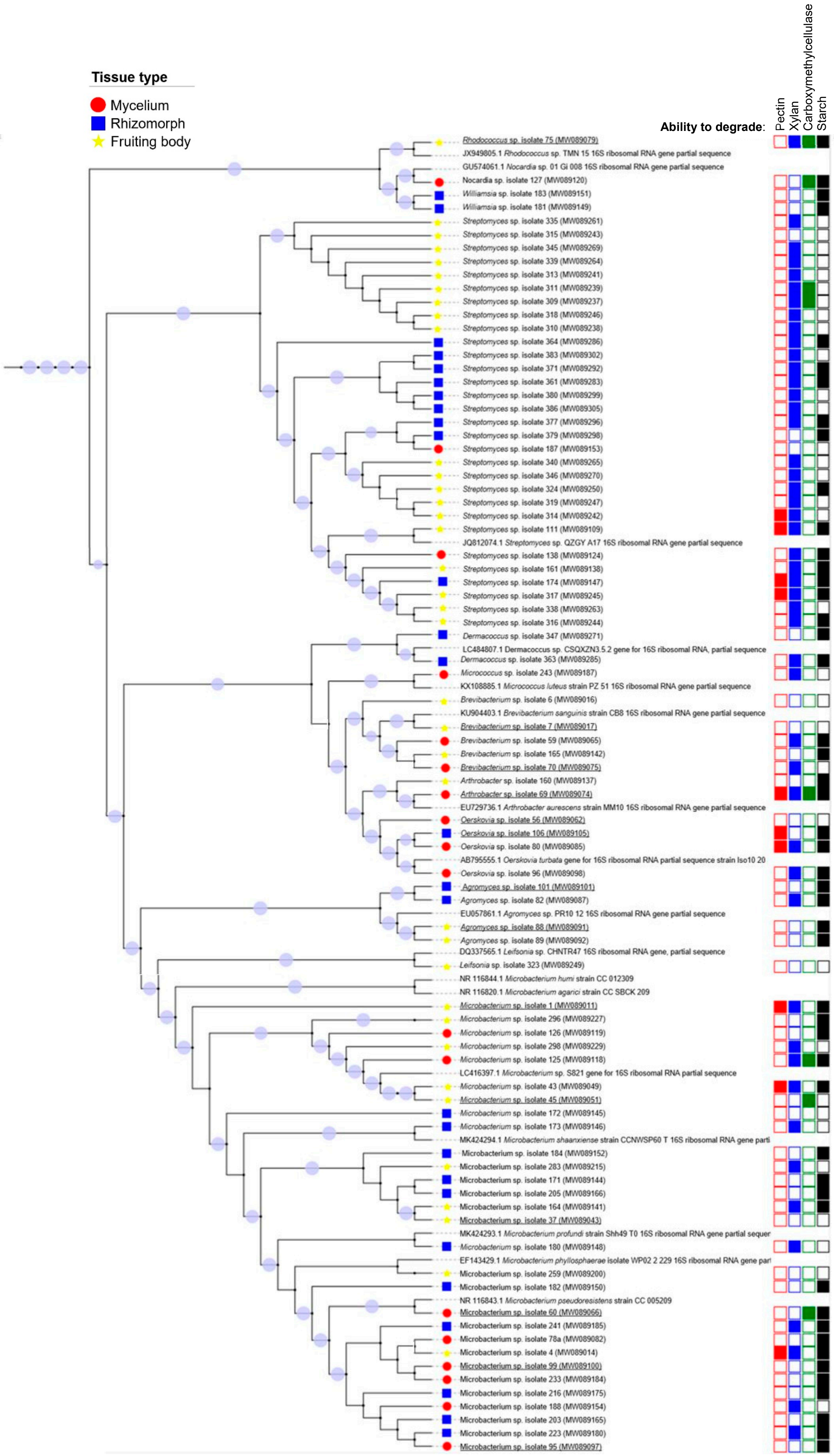

Tissue type
Mycelium
Rhizomorph
Fruiting body
Ability to degrade:
Pectin
Xylan
Carboxymethylcellulase
Starch
Rhodococcus sp. isolate 75 (MW089079)
JX949805.1 Rhodococcus sp. TMN 15 16S ribosomal RNA gene partial sequence
GU574061.1 Nocardia sp. 01 Gi 008 16S ribosomal RNA gene partial sequence
Nocardia sp. isolate 127 (MW089120)
Williamsia sp. isolate 183 (MW089151)
Williamsia sp. isolate 181 (MW089149)
Streptomyces sp. isolate 335 (MW089261)
Streptomyces sp. isolate 315 (MW089243)
Streptomyces sp. isolate 345 (MW089269)
Streptomyces sp. isolate 339 (MW089264)
Streptomyces sp. isolate 313 (MW089241)
Streptomyces sp. isolate 311 (MW089239)
Streptomyces sp. isolate 309 (MW089237)
Streptomyces sp. isolate 318 (MW089246)
Streptomyces sp. isolate 310 (MW089238)
Streptomyces sp. isolate 364 (MW089286)
Streptomyces sp. isolate 383 (MW089302)
Streptomyces sp. isolate 371 (MW089292)
Streptomyces sp. isolate 361 (MW089283)
Streptomyces sp. isolate 380 (MW089299)
Streptomyces sp. isolate 386 (MW089305)
Streptomyces sp. isolate 377 (MW089296)
Streptomyces sp. isolate 379 (MW089298)
Streptomyces sp. isolate 187 (MW089153)
Streptomyces sp. isolate 340 (MW089265)
Streptomyces sp. isolate 346 (MW089270)
Streptomyces sp. isolate 324 (MW089250)
Streptomyces sp. isolate 319 (MW089247)
Streptomyces sp. isolate 314 (MW089242)
Streptomyces sp. isolate 111 (MW089109)
JQ812074.1 Streptomyces sp. QZGY A17 16S ribosomal RNA gene partial sequence
Streptomyces sp. isolate 138 (MW089124)
Streptomyces sp. isolate 161 (MW089138)
Streptomyces sp. isolate 174 (MW089147)
Streptomyces sp. isolate 317 (MW089245)
Streptomyces sp. isolate 338 (MW089263)
Streptomyces sp. isolate 316 (MW089244)
Dermacoccus sp. isolate 347 (MW089271)
LC484807.1 Dermacoccus sp. CSQXZN3.5.2 gene for 16S ribosomal RNA, partial sequence
Dermacoccus sp. isolate 363 (MW089285)
Micrococcus sp. isolate 243 (MW089187)
KX108885.1 Micrococcus luteus strain PZ 51 16S ribosomal RNA gene partial sequence
Brevibacterium sp. isolate 6 (MW089016)
KU904403.1 Brevibacterium sanguinis strain CB8 16S ribosomal RNA gene partial sequence
Brevibacterium sp. isolate 7 (MW089017)
Brevibacterium sp. isolate 59 (MW089065)
Brevibacterium sp. isolate 165 (MW089142)
Brevibacterium sp. isolate 70 (MW089075)
Arthrobacter sp. isolate 160 (MW089137)
Arthrobacter sp. isolate 69 (MW089074)
EU729736.1 Arthrobacter aurescens strain MM10 16S ribosomal RNA gene partial sequence
Oerskovia sp. isolate 56 (MW089062)
Oerskovia sp. isolate 106 (MW089105)
Oerskovia sp. isolate 80 (MW089085)
AB795555.1 Oerskovia turbata gene for 16S ribosomal RNA partial sequence strain Iso10 20
Oerskovia sp. isolate 96 (MW089098)
Agromyces sp. isolate 101 (MW089101)
Agromyces sp. isolate 82 (MW089087)
EU057861.1 Agromyces sp. PR10 12 16S ribosomal RNA gene partial sequence
Agromyces sp. isolate 88 (MW089091)
Agromyces sp. isolate 89 (MW089092)
DQ337565.1 Leifsonia sp. CHNTR47 16S ribosomal RNA gene, partial sequence
Leifsonia sp. isolate 323 (MW089249)
NR 116844.1 Microbacterium humi strain CC 012309
NR 116820.1 Microbacterium agarici strain CC SBCK 209
Microbacterium sp. isolate 1 (MW089011)
Microbacterium sp. isolate 296 (MW089227)
Microbacterium sp. isolate 126 (MW089119)
Microbacterium sp. isolate 298 (MW089229)
Microbacterium sp. isolate 125 (MW089118)
LC416397.1 Microbacterium sp. S821 gene for 16S ribosomal RNA partial sequence
Microbacterium sp. isolate 43 (MW089049)
Microbacterium sp. isolate 45 (MW089051)
Microbacterium sp. isolate 172 (MW089145)
Microbacterium sp. isolate 173 (MW089146)
MK424294.1 Microbacterium shaanxiense strain CCNWSP60 T 16S ribosomal RNA gene parti
Microbacterium sp. isolate 184 (MW089152)
Microbacterium sp. isolate 283 (MW089215)
Microbacterium sp. isolate 171 (MW089144)
Microbacterium sp. isolate 205 (MW089166)
Microbacterium sp. isolate 164 (MW089141)
Microbacterium sp. isolate 37 (MW089043)
MK424293.1 Microbacterium profundi strain Shh49 T0 16S ribosomal RNA gene partial sequer
Microbacterium sp. isolate 180 (MW089148)
EF143429.1 Microbacterium phyllosphaerae isolate WP02 2 229 16S ribosomal RNA gene part
Microbacterium sp. isolate 259 (MW089200)
Microbacterium sp. isolate 182 (MW089150)
NR 116843.1 Microbacterium pseudoresistens strain CC 005209
Microbacterium sp. isolate 60 (MW089066)
Microbacterium sp. isolate 241 (MW089185)
Microbacterium sp. isolate 78a (MW089082)
Microbacterium sp. isolate 4 (MW089014)
Microbacterium sp. isolate 99 (MW089100)
Microbacterium sp. isolate 233 (MW089184)
Microbacterium sp. isolate 216 (MW089175)
Microbacterium sp. isolate 188 (MW089154)
Microbacterium sp. isolate 203 (MW089165)
Microbacterium sp. isolate 223 (MW089180)
Microbacterium sp. isolate 95 (MW089097)

Figure S7 | Cladogram of bacteria belonging to the phylum Actinobacteria (PHYML). 16S rRNA gene sequences show the relationships between bacteria isolated from *S. lacrymans* tissue types. Most 16S rRNA gene sequences had high similarity with type species (>98%-100%); final assignment at genera level was based on the best blast result and the phylogenetic tree. 99 sequences were used for tree creation. Light blue circles indicate a chi^2 statistics greater than 0.75. Reference sequences were retrieved from NCBI. Symbols that indicate tissue of isolation: red circle = bacteria isolated from mycelium; star = bacteria isolated from fruiting body and blue rectangle = bacteria isolated from rhizomorphs. Symbols on the right: red rectangle indicates ability to degrade pectin, blue rectangle indicates ability to degrade xylan, green rectangle indicates ability to degrade carboxymethylcellulose and black rectangle indicates ability to degrade starch. Underlined isolates were tested in co-culture assays.

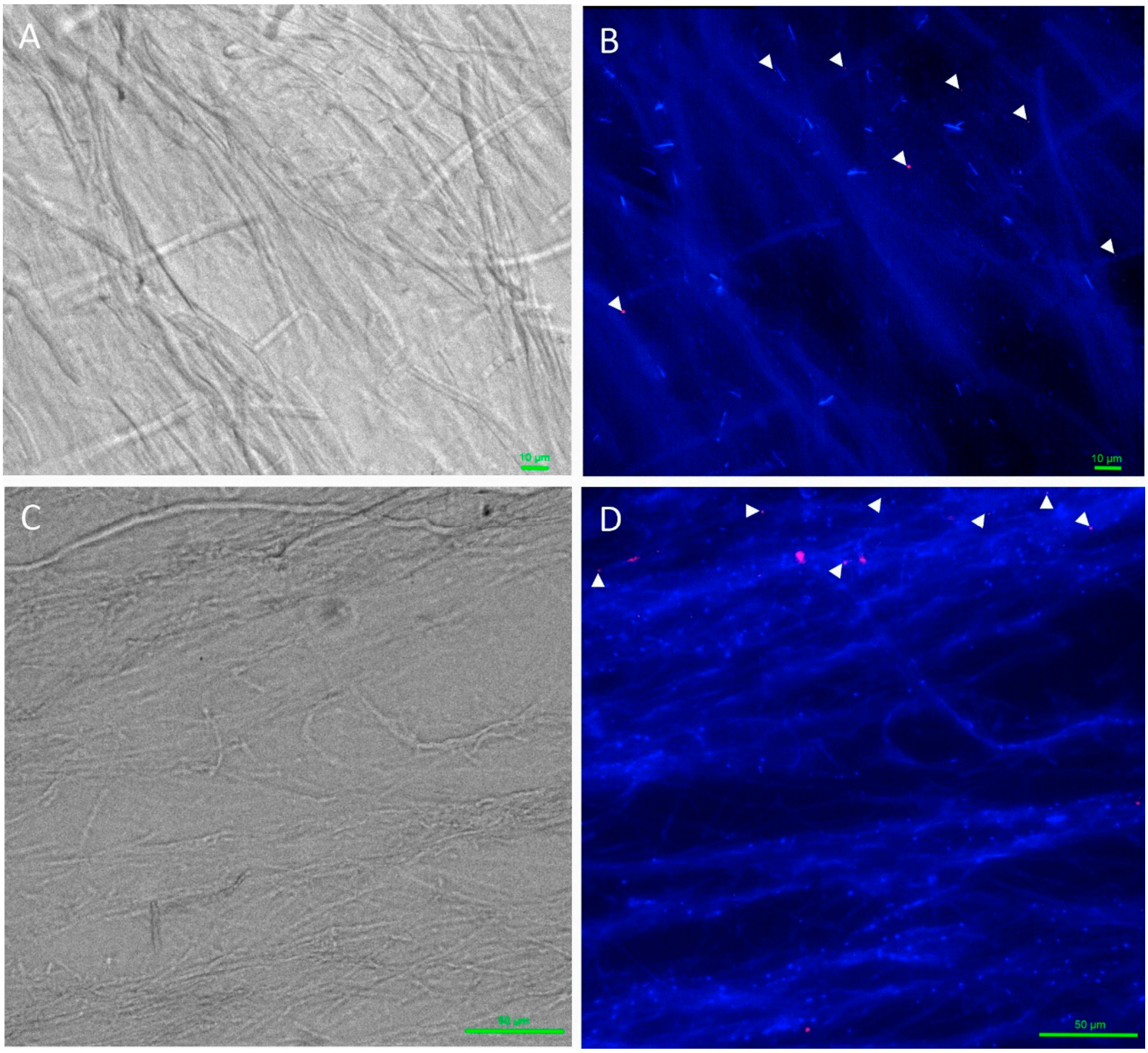

Figure S8 | Localization of bacteria on *S. lacrymans* rhizomorphs and mycelium. A Overlay of signals derived from EUB338 Mix-Cy5 and DAPI. Investigated tissue type = rhizomorph. **B** Transmitted light picture of rhizomorph. **C** Overlay of signals derived from EUB338 Mix-Cy5 and DAPI. Investigated tissue type = mycelium. **D** Transmitted light picture of fungal mycelium. The general bacterial EUB338 Mix probe gave red signals. Signals are highlighted with white arrows. Scale bars are 10 and 50 µm.

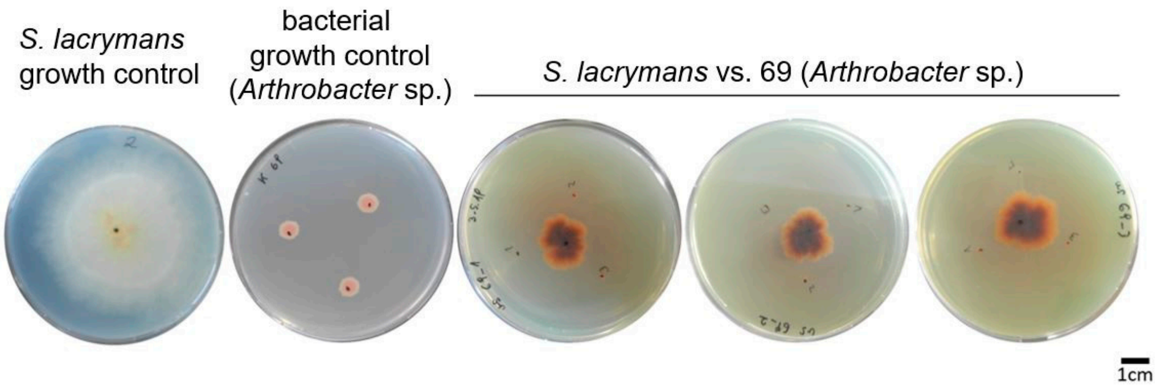

Figure S9 | Inhibition of bacterial growth by *S. lacrymans*. Growth impairment of bacterial isolate 69 (*Arthrobacter* sp.) during co-cultivation with *S. lacrymans*. Pictures were taken after cultivation for 31 days on minimal media at 25 °C.

Table S1: Accession numbers for sequences of bacterial isolates retrieved from NCBI: Bacteria isolated from *Serpula lacrymans* or *Coniophora puteana* (CP) tissue (FB = Fruiting body, M = Mycelia, R = Rhizomorph) on either Tryptone Soy agar (TSA), R2A or Malt extract agar (MEA); + means sequences are included in the cladograms or – no consideration in the phylogenetic analysis.

Accession No.	Isolate No.	Species	isolation		included in Cladogram
			tissue	media	
MW089011	1	*Microbacterium* sp.	FB	TSA	+
MW089012	2	*Microbacterium* sp.	FB	TSA	-
MW089013	3	*Staphylococcus* sp.	FB	TSA	+
MW089014	4	*Microbacterium* sp.	FB	TSA	+
MW089015	5	*Rahnella* sp.	FB	TSA	+
MW089016	6	*Brevibacterium* sp.	FB	TSA	+
MW089017	7	*Brevibacterium* sp.	FB	TSA	+
MW089018	8	*Microbacterium* sp.	M	TSA	-
MW089019	9	*Staphylococcus* sp.	M	TSA	+
MW089020	10	*Staphylococcus* sp.	M	TSA	+
MW089021	11	*Stenotrophomonas* sp.	M	TSA	+
MW089022	12	*Raoultella* sp.	M	TSA	+
MW089023	13	*Serratia* sp.	M	TSA	+
MW089024	15	*Sphingobacterium* sp.	M	TSA	+
MW089025	16	*Raoultella* sp.	M	TSA	+
MW089026	17	*Okibacterium* sp.	M	TSA	-
MW089027	18	*Ewingella* sp.	M	TSA	+
MW089028	20	*Microbacterium* sp.	FB CP	TSA	-
MW089029	21	*Pseudomonas* sp.	FB CP	TSA	-
MW089030	22	*Microbacterium* sp.	FB CP	TSA	-
MW089031	23	*Microbacterium* sp.	FB CP	TSA	-
MW089032	24	*Microbacterium* sp.	FB CP	TSA	-
MW089033	26	*Paenibacillus* sp.	M CP	TSA	-
MW089034	27	*Bacillus* sp.	M CP	TSA	-
MW089035	28	*Advenella* sp.	M CP	TSA	-
MW089036	29	*Pseudomonas* sp.	M CP	TSA	-
MW089037	30	*Bacillus* sp.	M CP	TSA	-
MW089038	31	*Staphylococcus* sp.	M CP	TSA	-
MW089039	33	*Sporosarcina* sp.	M CP	TSA	-
MW089040	34	*Bacillus* sp.	M CP	TSA	-
MW089041	35	*Stenotrophomonas* sp.	FB	TSA	+
MW089042	36	*Paenibacillus* sp.	FB	TSA	+
MW089043	37	*Microbacterium* sp.	FB	TSA	+
MW089044	38	*Stenotrophomonas* sp.	FB	TSA	+

MW089045	39	*Pseudomonas* sp.	FB	TSA	+
MW089046	40	*Staphylococcus* sp.	FB	TSA	+
MW089047	41	*Flavobacterium* sp.	FB	TSA	+
MW089048	42	*Pseudomonas* sp.	FB	TSA	+
MW089049	43	*Microbacterium* sp.	FB	TSA	+
MW089050	44	*Paenisporosarcina* sp.	FB	TSA	+
MW089051	45	*Microbacterium* sp.	FB	TSA	+
MW089052	46	*Flavobacterium* sp.	FB	TSA	+
MW089053	47	*Bacillus* sp.	R	TSA	-
MW089054	48	*Paenibacillus* sp.	R	TSA	+
MW089055	49	*Advenella* sp.	R	TSA	+
MW089056	50	*Bordetella* sp.	R	TSA	+
MW089057	51	*Pseudomonas* sp.	R	TSA	+
MW089058	52	*Alcaligenaceae bacterium*	R	TSA	+
MW089059	53	*Stenotrophomonas* sp.	M	TSA	+
MW089060	54	*Bacillus* sp.	M	TSA	+
MW089061	55	*Pseudomonas* sp.	M	TSA	+
MW089062	56	*Oerskovia* sp.	M	TSA	+
MW089063	57	*Advenella* sp.	M	TSA	+
MW089064	58	*Bordetella* sp.	M	TSA	+
MW089065	59	*Brevibacterium* sp.	M	TSA	+
MW089066	60	*Microbacterium* sp.	M	TSA	+
MW089067	61	*Stenotrophomonas* sp.	M	TSA	+
MW089068	62	*Raoultella* sp.	M	TSA	+
MW089069	63	*Paenibacillus* sp.	M	TSA	+
MW089070	65	*Alcaligenes* sp.	M	TSA	+
MW089071	66	*Stenotrophomonas* sp.	M	TSA	+
MW089072	67	*Staphylococcus* sp.	M	TSA	+
MW089073	68	*Staphylococcus* sp.	M	TSA	+
MW089074	69	*Arthrobacter* sp.	M	TSA	+
MW089075	70	*Brevibacterium* sp.	M	TSA	+
MW089076	71	*Bacillus* sp.	FB	TSA	+
MW089077	72	*Sporosarcina* sp.	FB	TSA	+
MW089078	73	*Pseudomonas* sp.	FB	TSA	+
MW089079	75	*Rhodococcus* sp.	FB	TSA	+
MW089080	76a	*Sporosarcina* sp.	FB	TSA	+
MW089081	76b	*Sporosarcina* sp.	FB	TSA	+
MW089082	78a	*Microbacterium* sp.	M	TSA	+
MW089083	78b	*Pseudomonas* sp.	M	TSA	+
MW089084	79	*Paenibacillus* sp.	M	TSA	-
MW089085	80	*Oerskovia* sp.	M	TSA	+
MW089086	81	*Erwinia* sp.	M	TSA	+
MW089087	82	*Agromyces* sp.	R	TSA	+
MW089088	84	*Bacillus* sp.	R	TSA	+

MW089089	86	*Bacillus* sp.	R	TSA	+
MW089090	87	*Bacillus* sp.	R	TSA	+
MW089091	88	*Agromyces* sp.	FB	TSA	+
MW089092	89	*Agromyces* sp.	FB	TSA	+
MW089093	90	*Bacillus* sp.	FB	TSA	+
MW089094	91	*Pseudomonas* sp.	FB	R2A	+
MW089095	92	*Bacillus* sp.	FB	R2A	+
MW089096	93	*Streptomyces* sp.	FB	R2A	-
MW089097	95	*Microbacterium* sp.	M	R2A	+
MW089098	96	*Oerskovia* sp.	M	R2A	+
MW089099	98	*Bacillus* sp.	M	R2A	+
MW089100	99	*Microbacterium* sp.	M	R2A	+
MW089101	101	*Agromyces* sp.	R	R2A	+
MW089102	102	*Bacillus* sp.	R	R2A	+
MW089103	104	*Bacillus* sp.	R	R2A	+
MW089104	105	*Chryseobacterium* sp.	R	R2A	+
MW089105	106	*Oerskovia* sp.	R	R2A	+
MW089106	107	*Bacillus* sp.	FB	R2A	+
MW089107	108	*Bacillus* sp.	FB	R2A	+
MW089108	109	*Caballeronia* sp.	FB	R2A	+
MW089109	111	*Streptomyces* sp.	FB	R2A	+
MW089110	114	*Solibacillus* sp.	FB	TSA	+
MW089111	115	*Lysobacter* sp.	FB	TSA	+
MW089112	116	*Paraburkholderia* sp.	FB	R2A	+
MW089113	117	*Bacillus* sp.	M	TSA	+
MW089114	118	*Flavobacterium* sp.	M	TSA	+
MW089115	119	*Flavobacterium* sp.	M	TSA	+
MW089116	120	*Bacillus* sp.	M	TSA	+
MW089117	124	*Paucisalibacillus* sp.	M	TSA	+
MW089118	125	*Microbacterium* sp.	M	TSA	+
MW089119	126	*Microbacterium* sp.	M	TSA	+
MW089120	127	*Nocardia* sp.	M	TSA	+
MW089121	129	*Pseudomonas* sp.	M	MEA	+
MW089122	134	*Enterobacter* sp.	M	TSA	+
MW089123	135	*Olivibacter* sp.	M	TSA	+
MW089124	138	*Streptomyces* sp.	M	TSA	+
MW089125	142	*Enterobacter* sp.	M	MEA	+
MW089126	143	*Pseudomonas* sp.	M	TSA	+
MW089127	144	*Pseudomonas* sp.	M	TSA	+
MW089128	145	*Bacillus* sp.	M	TSA	+
MW089129	146	*Achromobacter* sp.	M	TSA	+
MW089130	148	*Bacillus* sp.	M	TSA	+
MW089131	149	*Stenotrophomonas* sp.	M	TSA	+
MW089132	150	*Pseudomonas* sp.	M	TSA	+
MW089133	152	*Pseudomonas* sp.	M	R2A	-

MW089134	153	*Luteibacter* sp.	M	R2A	+
MW089135	157	*Bacillus* sp.	M	MEA	+
MW089136	158	*Bacillus* sp.	M	MEA	+
MW089137	160	*Arthrobacter* sp.	FB	TSA	+
MW089138	161	*Streptomyces* sp.	FB	TSA	+
MW089139	162	*Stenotrophomonas* sp.	FB	TSA	+
MW089140	163	*Advenella* sp.	FB	TSA	+
MW089141	164	*Microbacterium* sp.	FB	TSA	+
MW089142	165	*Brevibacterium* sp.	FB	TSA	+
MW089143	170	*Klebsiella* sp.	R	TSA	+
MW089144	171	*Microbacterium* sp.	R	TSA	+
MW089145	172	*Microbacterium* sp.	R	TSA	+
MW089146	173	*Microbacterium* sp.	R	TSA	+
MW089147	174	*Streptomyces* sp.	R	TSA	+
MW089148	180	*Microbacterium* sp.	R	R2A	+
MW089149	181	*Williamsia* sp.	R	R2A	+
MW089150	182	*Microbacterium* sp.	R	R2A	+
MW089151	183	*Williamsia* sp.	R	R2A	+
MW089152	184	*Microbacterium* sp.	M	R2A	+
MW089153	187	*Streptomyces* sp.	M	R2A	+
MW089154	188	*Microbacterium* sp.	M	TSA	+
MW089155	189	*Ochrobactrum* sp.	M	TSA	+
MW089156	190	*Ochrobactrum* sp.	M	TSA	+
MW089157	191	*Ochrobactrum* sp.	M	TSA	+
MW089158	192	*Ochrobactrum* sp.	M	TSA	+
MW089159	195	*Ochrobactrum* sp.	M	R2A	+
MW089160	196	*Ochrobactrum* sp.	M	R2A	+
MW089161	197	*Ochrobactrum* sp.	M	R2A	+
MW089162	199	*Pseudomonas* sp.	FB	TSA	+
MW089163	200	*Enterobacter* sp.	FB	TSA	+
MW089164	202	*Enterobacter* sp.	FB	TSA	+
MW089165	203	*Microbacterium* sp.	R	TSA	+
MW089166	205	*Microbacterium* sp.	R	TSA	+
MW089167	206	*Bacillus* sp.	R	TSA	+
MW089168	207	*Bacillus* sp.	R	TSA	+
MW089169	208	*Bacillus* sp.	R	TSA	+
MW089170	209	*Enterobacter* sp.	FB	MEA	+
MW089171	210	*Erwinia* sp.	FB	MEA	+
MW089172	212	*Enterobacter* sp.	FB	MEA	+
MW089173	213	*Bacillus* sp.	R	MEA	+
MW089174	215	*Pseudomonas* sp.	R	MEA	+
MW089175	216	*Microbacterium* sp.	R	MEA	+
MW089176	217	*Sphingobacterium* sp.	R	MEA	+
MW089177	219	*Pseudomonas* sp.	R	MEA	+
MW089178	221	*Pseudomonas* sp.	R	R2A	+

MW089179	222	*Pseudomonas* sp.	R	R2A	+
MW089180	223	*Microbacterium* sp.	R	R2A	+
MW089181	227	*Pseudomonas* sp.	R	TSA	+
MW089182	229	*Pseudomonas* sp.	R	TSA	+
MW089183	231	*Pseudomonas* sp.	M	TSA	+
MW089184	233	*Microbacterium* sp.	M	TSA	+
MW089185	241	*Microbacterium* sp.	R	R2A	+
MW089186	242	*Pseudomonas* sp.	M	R2A	+
MW089187	243	*Micrococcus* sp.	M	R2A	+
MW089188	244	*Pseudomonas* sp.	M	R2A	+
MW089189	246	*Bacillus* sp.	FB	R2A	+
MW089190	248	*Klebsiella* sp.	FB	R2A	+
MW089191	250	*Kluyvera* sp.	FB	R2A	+
MW089192	251	*Kluyvera* sp.	FB	R2A	+
MW089193	252	*Klebsiella* sp.	FB	R2A	+
MW089194	253	*Bacillus* sp.	FB	TSA	+
MW089195	254	*Bacillus* sp.	FB	TSA	+
MW089196	255	*Pseudomonas* sp.	FB	TSA	+
MW089197	256	*Pseudomonas* sp.	FB	TSA	+
MW089198	257	*Pseudomonas* sp.	FB	TSA	+
MW089199	258	*Phyllobacterium* sp.	FB	TSA	+
MW089200	259	*Microbacterium* sp.	FB	TSA	+
MW089201	260	*Bacillus* sp.	FB	TSA	+
MW089202	261	*Bacillus* sp.	M	TSA	+
MW089203	263	*Bacillus* sp.	M	TSA	+
MW089204	264	*Bacillus* sp.	M	TSA	+
MW089205	266	*Bacillus* sp.	M	MEA	+
MW089206	274	*Bacillus* sp.	FB	R2A	+
MW089207	275	*Bacillus* sp.	FB	R2A	+
MW089208	276	*Paenibacillus* sp.	FB	R2A	+
MW089209	277	*Pseudomonas* sp.	FB	R2A	+
MW089210	278	*Pseudomonas* sp.	FB	R2A	+
MW089211	279	*Bacillus* sp.	M	R2A	+
MW089212	280	*Bacillus* sp.	M	R2A	+
MW089213	281	*Serratia* sp.	M	R2A	+
MW089214	282	*Staphylococcus* sp.	FB	TSA	+
MW089215	283	*Microbacterium* sp.	FB	TSA	+
MW089216	284	*Staphylococcus* sp.	FB	TSA	+
MW089217	285	*Staphylococcus* sp.	FB	TSA	+
MW089218	286	*Bacillus* sp.	FB	TSA	+
MW089219	288	*Bacillus* sp.	FB	TSA	+
MW089220	289	*Bacillus* sp.	FB	TSA	+
MW089221	290	*Bacillus* sp.	FB	TSA	+
MW089222	291	*Staphylococcus* sp.	FB	TSA	+
MW089223	292	*Staphylococcus* sp.	FB	TSA	+

MW089224	293	*Bacillus* sp.	FB	R2A	+
MW089225	294	*Staphylococcus* sp.	FB	R2A	+
MW089226	295	*Staphylococcus* sp.	FB	R2A	+
MW089227	296	*Microbacterium* sp.	FB	R2A	+
MW089228	297	*Staphylococcus* sp.	FB	R2A	+
MW089229	298	*Microbacterium* sp.	FB	R2A	+
MW089230	299	*Staphylococcus* sp.	FB	MEA	+
MW089231	301	*Staphylococcus* sp.	FB	MEA	+
MW089232	302	*Staphylococcus* sp.	FB	MEA	+
MW089233	305	*Ewingella* sp.	FB	TSA	+
MW089234	306	*Ewingella* sp.	FB	TSA	+
MW089235	307	*Ewingella* sp.	FB	TSA	+
MW089236	308	*Ewingella* sp.	FB	TSA	+
MW089237	309	*Streptomyces* sp.	FB	TSA	+
MW089238	310	*Streptomyces* sp.	FB	TSA	+
MW089239	311	*Streptomyces* sp.	FB	TSA	+
MW089240	312	*Ewingella* sp.	FB	TSA	+
MW089241	313	*Streptomyces* sp.	FB	TSA	+
MW089242	314	*Streptomyces* sp.	FB	TSA	+
MW089243	315	*Streptomyces* sp.	FB	TSA	+
MW089244	316	*Streptomyces* sp.	FB	TSA	+
MW089245	317	*Streptomyces* sp.	FB	TSA	+
MW089246	318	*Streptomyces* sp.	FB	TSA	-
MW089247	319	*Streptomyces* sp.	FB	TSA	+
MW089248	320	*Ewingella* sp.	FB	TSA	+
MW089249	323	*Leifsonia* sp.	FB	TSA	+
MW089250	324	*Streptomyces* sp.	FB	TSA	+
MW089251	325	*Ewingella* sp.	FB	R2A	+
MW089252	326	*Ewingella* sp.	FB	R2A	+
MW089253	327	*Ewingella* sp.	FB	R2A	+
MW089254	328	*Ewingella* sp.	FB	R2A	+
MW089255	329	*Ewingella* sp.	FB	R2A	+
MW089256	330	*Ewingella* sp.	FB	R2A	+
MW089257	331	*Ewingella* sp.	FB	R2A	+
MW089258	332	*Ewingella* sp.	FB	R2A	+
MW089259	333	*Ewingella* sp.	FB	R2A	+
MW089260	334	*Ewingella* sp.	FB	R2A	+
MW089261	335	*Streptomyces* sp.	FB	R2A	+
MW089262	337	*Moraxella* sp.	FB	R2A	+
MW089263	338	*Streptomyces* sp.	FB	R2A	+
MW089264	339	*Streptomyces* sp.	FB	R2A	+
MW089265	340	*Streptomyces* sp.	FB	R2A	+
MW089266	341	*Ewingella* sp.	FB	R2A	+
MW089267	343	*Burkholderia* sp.	FB	R2A	+
MW089268	344	*Ewingella* sp.	FB	R2A	+

MW089269	345	*Streptomyces* sp.	FB	R2A	+
MW089270	346	*Streptomyces* sp.	FB	R2A	+
MW089271	347	*Dermacoccus* sp.	R	MEA	+
MW089272	348	*Enterobacter* sp.	FB	MEA	+
MW089273	349	*Enterobacter* sp.	FB	MEA	+
MW089274	350	*Enterobacter* sp.	FB	MEA	+
MW089275	351	*Klebsiella* sp.	FB	MEA	+
MW089276	352	*Klebsiella* sp.	FB	MEA	-
MW089277	353	*Staphylococcus* sp.	R	TSA	-
MW089278	355	*Staphylococcus* sp.	R	TSA	+
MW089279	356	*Staphylococcus* sp.	R	TSA	+
MW089280	358	*Staphylococcus* sp.	R	TSA	+
MW089281	359	*Staphylococcus* sp.	R	TSA	+
MW089282	360	*Staphylococcus* sp.	R	TSA	+
MW089283	361	*Streptomyces* sp.	R	TSA	+
MW089284	362	*Staphylococcus* sp.	R	TSA	+
MW089285	363	*Dermacoccus* sp.	R	TSA	+
MW089286	364	*Streptomyces* sp.	R	TSA	+
MW089287	365	*Staphylococcus* sp.	R	TSA	+
MW089288	367	*Bacillus* sp.	R	TSA	+
MW089289	368	*Staphylococcus* sp.	R	TSA	+
MW089290	369	*Bacillus* sp.	R	TSA	+
MW089291	370	*Staphylococcus* sp.	R	TSA	+
MW089292	371	*Streptomyces* sp.	R	TSA	+
MW089293	372	*Staphylococcus* sp.	R	TSA	+
MW089294	373	*Staphylococcus* sp.	R	TSA	+
MW089295	374	*Paenisporosarcina* sp.	R	TSA	+
MW089296	377	*Streptomyces* sp.	R	R2A	+
MW089297	378	*Staphylococcus* sp.	R	R2A	+
MW089298	379	*Streptomyces* sp.	R	R2A	+
MW089299	380	*Streptomyces* sp.	R	R2A	+
MW089300	381	*Staphylococcus* sp.	R	R2A	+
MW089301	382	*Staphylococcus* sp.	R	R2A	+
MW089302	383	*Streptomyces* sp.	R	R2A	+
MW089303	384	*Staphylococcus* sp.	R	R2A	+
MW089304	385	*Staphylococcus* sp.	R	R2A	+
MW089305	386	*Streptomyces* sp.	R	R2A	+

Table S2: Estimated abundances of bacterial species derived from different tissues of *S. lacrymans* (see Fig. 2). Abundances of bacterial species (in CFU g^{-1}) were estimated by an inverse morphotype approach. Because of the reciprocal approach, only relative abundances are indicated. Bacterial abundances of all used isolation media were summarized.

**Genera**	**Tissue type**			**Sum [CFU g^{-1}; %]**
	Fruiting body	Mycelium	Rhizomorph	
Paenibacillus sp.	80720	1008.2	4448.4	
Sporosarcina sp.	4023216	-	-	
Paeni*sporosarcina* sp.	1757400000	-	6081.9	
Solibacillus sp.	361800	-	-	
Bacillus sp.	1914474399	5871251379	71259161.5	
Pausicalibacillus sp.	-	385920000	-	
Staphylococcus sp.	1034950852	2414403.6	506825	
**Firmicutes spp.**	**4711290988**	**6281316791**	**71776516.8**	**11064384295; 52%**
Microbacterium spp.	130853242.9	123524399	6320480	
Agromyces spp.	217482	-	41653200	
Oerskovia spp.	-	195548222	-	
Cellulomonas spp.	-	161.36	-	
Arhtrobacter spp.	-	242.16	-	
Brevibacterium spp.	36340637.4	-	-	
Streptomyces spp.	331878384	16040000	111294715.4	
Rhodococcus spp.	4422	-	-	
Nocardia spp.	-	4020000	-	
Micrococcus spp.	n/a	n/a	n/a	
Leifsonia spp.	2032	-	-	
Dermacoccus spp.	202730000	-	-	
**Actinobacteria spp.**	**702026200.3**	**339133024.56**	**159268395.4**	**1200427620; 6%**
Flavobacterium spp.	769216000	40228140	-	
Sphingobacterium spp.	-	407.6	-	
Chryseobacterium spp.	n/a	n/a	n/a	
Pseudosphingobacterium spp.	n/a	n/a	n/a	
***Bacteroidetes spp*.**	**769216000**	**40228547.6**	**0**	**809444547.6; 4%**
Stenotrophomonas spp.	166283.2	185483.64	2830800	
Bordetella spp.	-	80440	1124232000	
Alcaligenes spp.	-	161.36	6066	
Advenella spp.	-	804400	29925600	

Pseudomonas spp.	54622374	2183356548	1161025013	
Erwinia spp.	16800000	40360000	-	
Rahnella spp.	484,488	-	-	
Ewingella spp.	1538224	89672000	-	
Pantoea spp.	24120	-	-	
Raoultella spp.	-	1176,08	-	
Serratia spp.	-	247418581.52	24696	
Achromobacter spp.	-	103300000	-	
Burkholderia spp.	3341984	-	-	
Enterobacter spp.	18043920	-	-	
Klebsiella spp.	7190172	40450000	303400	
Luteibacter spp.	-	82.64	-	
Ochrobactrum spp.	-	99851500.8	-	
Xanthomonas spp.	n/a	n/a	n/a	
Pectobacterium spp.	137182.4	-	-	
Kluyvera spp.	2688000000	-	-	
Phyllobacterium spp.	4080000	-	-	
Moraxella spp.	125984	-	-	
***Proteobacteria spp*.**	**2794070728**	**2805480374**	**2318347575**	**7917898677; 38%**
**Total [CFU g^{-1} tissue]**	**8976477932**	**9466158737**	**2549392487**	**20992029156**
**[%]**	**42.76**	**45.1**	**12.144**	**100**

Table S3: Results of Pectinase, Xylanase, Cellulase and Amylase Production Assays: Bacteria isolated from *Serpula lacrymans* tissue (FB = Fruiting body, M = Mycelia, R = Rhizomorph); - means no production of according enzyme (i.e. no halo), ~ minimal production of enzyme (halo zone not more than 1 mm of the edges of the colonies), + enzymatic activity (halo zone smaller than 8 mm from the edges of the colonies), and ++ high enzymatic activity (halo zone larger than 8 mm). Plates were evaluated after 2 to 4 days (25 °C). * Bacterial isolate could not be identified based on the phylogenetic analysis.

			Enzyme produced			
**Isolate No.**	**Species**	**tissue of Isolation**	**Pectinase**	**Xylanase**	**Cellulase**	**Amylase**
01	*Microbacterium* sp.	FB	~	+	-	+
02	*Microbacterium* sp.	FB	~	-	-	-
03	*Staphylococcus* sp.	FB	~	-	-	-
04	*Microbacterium* sp.	FB	~	-	-	+
05	*Rahnella* sp.	FB	-	~	+	-
06	*Brevibacterium* sp.	FB	-	-	-	-
07	*Brevibacterium* sp.	FB	-	-	-	-
08	*Microbacterium* sp.	M	-	-	-	+
09	*Staphylococcus* sp.	M	-	-	-	-
10	*Staphylococcus* sp.	M	-	+	+	+
11	*Stenotrophomonas* sp.	M	-	-	-	-
12	*Raoultella* sp.	M	-	-	~	~
13	*Serratia* sp.	M	+	+	+	+
14	*Pseudomonas* sp.	M	+	-	++	+
15	*Sphingobacterium* sp.	M	-	-	-	+
16	*Raoultella* sp.	M	-	+	-	+
17	*Okibacterium* sp.	M	-	-	-	+
18	*Ewingella* sp.	M	+	-	-	+
19	*Paenibacillus* sp.	FB	-	-	-	+
20	*Microbacterium* sp.	FB	-	+	-	+
21	*Pseudomonas* sp.	FB	-	-	-	+
22	*Microbacterium* sp.	FB	+	-	-	+
23	*Microbacterium* sp.	FB	-	+	-	-
24	*Microbacterium* sp.	FB	-	-	~	-
25	*Psychrobacillus* sp.	M	-	+	-	+
26	*Paenibacillus* sp.	M	+	-	-	+
27	*Bacillus* sp.	M	+	-	-	+
28	*Advenella* sp.	M	-	-	-	-
29	*Pseudomonas* sp.	M	-	-	-	+
30	*Bacillus* sp.	M	+	-	-	+

31	*Staphylococcus* sp.	M	-	-	-	-
32	*Serratia* sp.	M	-	+	-	-
33	*Sporosarcina* sp.	M	-	-	-	-
34	*Bacillus* sp.	M	-	+	+	+
35	*Stenotrophomonas* sp.	FB	-	-	-	-
36	*Paenibacillus* sp.	FB	+	+	+	+
37	*Microbacterium* sp.	FB	-	-	-	-
38	*Stenotrophomonas* sp.	FB	-	-	-	-
39	*Pseudomonas* sp.	FB	~	-	+	-
40	*Staphylococcus* sp.	FB	-	-	-	-
41	*Flavobacterium* sp.	FB	+	+	+	+
42	*Pseudomonas* sp.	FB	-	-	-	+
43	*Microbacterium* sp.	FB	+	+	-	+
44	*Paenisporosarcina* sp.	FB	~	-	-	-
45	*Microbacterium* sp.	FB	-	-	+	-
46	*Flavobacterium* sp.	FB	+	+	+	+
47	*Bacillus* sp.	R	~	-	+	-
48	*Paenibacillus* sp.	R	+	-	-	+
49	*Advenella* sp.	R	-	~	+	+
50	*Bordetella* sp.	R	-	-	-	+
51	*Pseudomonas* sp.	R	-	+	~	+
52	*Alcaligenaceae* bacterium	R	-	-	-	-
53	*Stenotrophomonas* sp.	M	-	+	+	+
54	*Bacillus* sp.	M	-	-	~	-
55	*Pseudomonas* sp.	M	-	+	+	+
56	*Oerskovia* sp.	M	-	-	-	-
57	*Advenella* sp.	M	+	-	-	+
58	*Bordetella* sp.	M	-	-	+	-
59	*Brevibacterium* sp.	M	-	+	-	+
60	*Microbacterium* sp.	M	-	-	+	+
61	*Stenotrophomonas* sp.	M	-	-	-	~
62	*Raoultella* sp.	M	-	+	-	+
63	*Paenibacillus* sp.	M	+	-	-	+
64	*Cellulomonas* sp.	M	-	+	-	+
65	*Alcaligenaceae* sp.	M	-	+	-	+
66	*Stenotrophomonas* sp.	M	-	+	-	+
67	*Staphylococcus* sp.	M	-	+	+	-
68	*Staphylococcus* sp.	M	~	-	~	+
69	*Arthrobacter* sp.	M	+	+	+	+
70	*Brevibacterium* sp.	M	-	+	-	
71	*Bacillus* sp.	FB	+	~	+	+
72	*Sporosarcina* sp.	FB	-	-	-	-
73	*Pseudomonas* sp.	FB	-	-	~	+
74	*Pantoea* sp.	FB	+	-	+	-
75	*Rhodococcus* sp.	FB	-	~	~	~

76a	*Sporosarcina* sp.	FB	-	-	-	~
77	*Serratia* sp.	M	+	+	-	+
78a	*Microbacterium* sp.	M	-	-	-	+
78b	*Pseudomonas* sp.	M	+	+	-	+
79	*Paenibacillus* sp.	M	+	+	+	+
80	*Oerskovia* sp.	M	+	+	-	+
81	*Erwinia* sp.	M	-	+	+	+
82	*Agromyces* sp.	R	-	+	-	+
83	*Bacillus* sp.	R	-	+	-	+
84	*Bacillus* sp.	R	-	-	-	++
85	*Bacillus* sp.	R	-	+	-	+
86	*Bacillus* sp.	R	-	-	+	+
87	*Bacillus* sp.	R	~	+	-	+
88	*Agromyces* sp.	FB	-	-	-	+
89	*Agromyces* sp.	FB	-	-	-	+
90	*Bacillus* sp.	FB	+	+	-	+
91	*Pseudomonas* sp.	FB	+	+	+	+
92	*Bacillus* sp.	FB	+	+	-	+
93	*Streptomyces* sp.	FB	-	+	-	+
94	*	FB	-	+	-	+
95	*Microbacterium* sp.	M	-	-	-	+
96	*Oerskovia* sp.	M	-	+	-	+
98	*Bacillus* sp.	M	+	+	-	+
99	*Microbacterium* sp.	M	-	-	-	+
101	*Agromyces* sp.	R	-	-	-	+
102	*Bacillus* sp.	R	+	+	-	++
104	*Bacillus* sp.	R	-	+	+	++
105	*Chryseobacterium* sp.	R	+	+	-	+
106	*Oerskovia* sp.	R	+	-	-	+
107	*Bacillus* sp.	FB	+	+	-	+
108	*Bacillus* sp.	FB	-	+	+	++
109	*Caballeronia* sp.	FB	-	+	-	+
111	*Streptomyces* sp.	FB	~	+	-	+
113	*Pichia/Saccharomyces*	FB	-	+	-	+
114	*Solibacillus* sp.	FB	-	+	-	-
115	*Lysobacter* sp.	FB	-	-	-	+
116	*Paraburkholderia* sp.	FB	-	+	-	+
117	*Bacillus* sp.	M	-	-	-	+
118	*Flavobacterium* sp.	M	-	-	-	++
119	*Flavobacterium* sp.	M	-	-	-	+
120	*Bacillus* sp.	M	-	+	-	-
121	*	M	-	-	-	-
124	*Paucisalibacillus* sp.	M	-	+	-	~
125	*Microbacterium* sp.	M	-	+	+	+
126	*Microbacterium* sp.	M	-	-	-	~

127	*Nocardia* sp.	M	-	-	~	++
129	*Pseudomonas* sp.	M	-	-	+	+
134	*Enterobacter* sp.	M	+	-	-	+
135	*Pseudosphingobacterium* sp.	M	-	-	++	++
136	*	M	-	-	-	+
138	*Streptomyces* sp.	M	-	+	-	++
142	*Enterobacter* sp.	M	-	-	-	~
143	*Pseudomonas* sp.	M	+	-	-	+
144	*Pseudomonas* sp.	M	-	-	-	+
145	*Bacillus* sp.	M	++	-	-	+
146	*Achromobacter* sp.	M	-	-	-	-
147	*	M	-	-	-	+
148	*Bacillus* sp.	M	-	+	-	+
149	*Stenotrophomonas* sp.	M	-	-	-	+
150	*Pseudomonas* sp.	M	-	-	-	-
151	*Bacillus* sp.	M	-	-	-	+
152	*Pseudomonas* sp.	M	+	-	-	+
153	*Luteibacter* sp.	M	-	-	-	+
154	*Candida* sp.	M	+	+	+	+
155	*Pseudomonas* sp.	M	++	-	-	+
157	*Bacillus* sp.	M	++	-	-	+
158	*Bacillus* sp.	M	-	+	-	+
160	*Arthrobacter* sp.	FB	-	-	-	+
161	*Streptomyces* sp.	FB	-	+	-	++
162	*Stenotrophomonas* sp.	FB	-	-	-	~
163	*Advenella* sp.	FB	-	-	+	-
164	*Microbacterium* sp.	FB	-	+	-	+
165	*Brevibacterium* sp.	FB	-	-	-	+
170	*Klebsiella* sp.	R	-	-	-	~
171	*Microbacterium* sp.	R	-	-	-	+
172	*Microbacterium* sp.	R	-	-	-	-
173	*Microbacterium* sp.	R	-	+	-	-
174	*Streptomyces* sp.	R	+	+	-	+
180	*Microbacterium* sp.	R	-	+	-	-
181	*Williamsia* sp.	R	-	-	-	+
182	*Microbacterium* sp.	R	-	-	-	+
183	*Williamsia* sp.	R	-	-	-	~
184	*Microbacterium* sp.	R	-	-	-	+
186	*	M	-	-	-	-
187	*Streptomyces* sp.	M	-	-	-	-
188	*Microbacterium* sp.	M	-	+	-	-
189	*Ochrobactrum* sp.	M	-	-	-	+
190	*Ochrobactrum* sp.	M	-	-	-	+
191	*Ochrobactrum* sp.	M	-	-	-	+

192	*Ochrobactrum* sp.	M	-	-	-	+
195	*Ochrobactrum* sp.	M	-	-	-	+
196	*Ochrobactrum* sp.	M	-	-	-	+
197	*Ochrobactrum* sp.	M	-	-	-	++
198	*	FB	-	+	+	-
199	*Pseudomonas* sp.	FB	-	-	+	+
200	*Enterobacter* sp.	FB	-	-	-	+
201	*Bacillus* sp.	FB	-	+	+	+
202	*Enterobacter* sp.	FB	-	+	-	+
203	*Microbacterium* sp.	R	-	-	-	+
204	*	R	+	*	-	+
205	*Microbacterium* sp.	R	-	-	-	++
206	*Bacillus* sp.	R	-	+	-	+
207	*Bacillus* sp.	R	-	+	-	+
208	*Bacillus* sp.	R	-	-	-	+
209	*Enterobacter* sp.	FB	-	-	-	+
210	*Erwinia* sp.	FB	-	+	~	+
211	*	FB	+	-	-	+
212	*Enterobacter* sp.	FB	-	+	-	+
213	*Bacillus* sp.	R	-	+	~	+
215	*Pseudomonas* sp.	R	-	-	+	+
216	*Microbacterium* sp.	R	-	-	-	+
217	*Sphingobacterium* sp.	R	-	-	-	+
218	*Stenotrophomonas* sp.	R	~	-	-	~
219	*Pseudomonas* sp.	R	+	-	-	+
220	*	R	-	+	-	~
221	*Pseudomonas* sp.	R	+	+	+	+
222	*Pseudomonas* sp.	R	+	-	-	+
223	*Microbacterium* sp.	R	-	+	-	+
224	*	FB	-	+	-	+
225	*	FB	-	-	+	+
226	*	FB	-	+	-	+
227	*Pseudomonas* sp.	R	+	-	-	+
228	*	R	+	+	-	+
229	*Pseudomonas* sp.	R	+	-	+	+
230	*	M	-	+	-	+
231	*Pseudomonas* sp.	M	+	-	-	+
232	*	M	-	+	-	+
233	*Microbacterium* sp.	M	-	-	-	+
234	*	FB	-	+	-	+
235	*Pectobacterium* sp.	FB	-	+	-	+
236	*	R	-	+	-	+
238	*	R	+	+	-	+
239	*	M	+	-	-	+
240	*Serratia* sp.	R	+	-	-	+

241	*Microbacterium* sp.	R	-	~	-	+
242	*Pseudomonas* sp.	M	+	-	+	+
243	*Micrococcus* sp.	M	-	+	-	-
244	*Pseudomonas* sp.	M	+	-	+	+
245	*	FB	+	-	-	+
246	*Bacillus* sp.	FB	+	+	-	+
247	*	FB	-	+	+	-
248	*Klebsiella* sp.	FB	-	-	-	+
249	*	FB	+	-	-	+
250	*Kluyvera* sp.	FB	+	-	-	+
251	*Kluyvera* sp.	FB	+	*	-	+
252	*Klebsiella* sp.	FB	-	-	-	+
253	*Bacillus* sp.	FB	+	-	-	++
254	*Bacillus* sp.	FB	++	+	-	+
255	*Pseudomonas* sp.	FB	-	-	+	+
256	*Pseudomonas* sp.	FB	-	-	-	-
257	*Pseudomonas* sp.	FB	-	+	-	+
258	*Phyllobacterium* sp.	FB	-	-	-	+
259	*Microbacterium* sp.	FB	-	-	-	-
260	*Bacillus* sp.	FB	+	-	+	-
261	*Bacillus* sp.	M	-	++	-	-
262	*Klebsiella* sp.	M	-	-	-	+
263	*Bacillus* sp.	M	-	+	+	+
264	*Bacillus* sp.	M	-	+	+	+
265	*Serratia* sp.	M	+	+	-	-
266	*Bacillus* sp.	FB	+	-	-	+
268	*	M	~	-	-	-
269	*	M	-	-	-	-
274	*Bacillus* sp.	FB	++	-	+	+
275	*Bacillus* sp.	FB	~	+	+	++
276	*Paenibacillus* sp.	FB	-	-	-	+
277	*Pseudomonas* sp.	FB	-	+	-	+
278	*Pseudomonas* sp.	FB	-	-	-	+
279	*Bacillus* sp.	M	++	+	+	+
280	*Bacillus* sp.	M	-	-	++	++
281	*Serratia* sp.	M	-	-	-	+
282	*Staphylococcus* sp.	FB	-	-	-	+
283	*Microbacterium* sp.	FB	-	+	-	-
284	*Staphylococcus* sp.	FB	-	+	-	-
285	*Staphylococcus* sp.	FB	-	+	-	-
286	*Bacillus* sp.	FB	+	+	-	++
287	*Bacillus* sp.	FB	-	+	~	+
288	*Bacillus* sp.	FB	-	-	-	-
289	*Bacillus* sp.	FB	-	+	-	+
290	*Bacillus* sp.	FB	-	-	-	+

291	*Staphylococcus* sp.	FB	-	-	-	-
292	*Staphylococcus* sp.	FB	-	-	+	+
293	*Bacillus* sp.	FB	-	++	+	+
294	*Staphylococcus* sp.	FB	-	-	+	+
295	*Staphylococcus* sp.	FB	-	-	-	+
296	*Microbacterium* sp.	FB	-	-	-	++
297	*Staphylococcus* sp.	FB	-	+	-	-
298	*Microbacterium* sp.	FB	-	++	-	-
299	*Staphylococcus* sp.	FB	-	++	+	-
301	*Staphylococcus* sp.	FB	-	+	-	+
302	*Staphylococcus* sp.	FB	-	++	+	-
305	*Ewingella* sp.	FB	~	-	+	+
306	*Ewingella* sp.	FB	+	~	-	+
307	*Ewingella* sp.	FB	+	-	-	+
308	*Ewingella* sp.	FB	+	-	-	+
309	*Streptomyces* sp.	FB	-	+	~	-
310	*Streptomyces* sp.	FB	-	+	-	-
311	*Streptomyces* sp.	FB	-	+	+	-
312	*Ewingella* sp.	FB	+	-	-	+
313	*Streptomyces* sp.	FB	-	+	-	-
314	*Streptomyces* sp.	FB	~	+	-	-
315	*Streptomyces* sp.	FB	-	-	-	-
316	*Streptomyces* sp.	FB	-	+	-	+
317	*Streptomyces* sp.	FB	+	+	-	+
318	*Streptomyces* sp.	FB	-	+	-	-
319	*Streptomyces* sp.	FB	-	+	-	-
320	*Ewingella* sp.	FB	+	-	-	++
321	*	FB	-	+	-	++
322	*	FB	-	-	-	-
323	*Leifsonia* sp.	FB	-	-	-	-
324	*Streptomyces* sp.	FB	-	+	-	++
325	*Ewingella* sp.	FB	+	-	-	~
326	*Ewingella* sp.	FB	+	-	-	~
327	*Ewingella* sp.	FB	+	-	-	+
328	*Ewingella* sp.	FB	+	-	-	+
329	*Ewingella* sp.	FB	+	-	-	+
330	*Ewingella* sp.	FB	+	-	-	+
331	*Ewingella* sp.	FB	+	+	-	+
332	*Ewingella* sp.	FB	~	+	-	+
333	*Ewingella* sp.	FB	+	-	-	+
334	*Ewingella* sp.	FB	+	-	-	+
335	*Streptomyces* sp.	FB	-	+	-	-
337	*Moraxella* sp.	FB	~	+	-	-
338	*Streptomyces* sp.	FB	-	+	-	-
339	*Streptomyces* sp.	FB	-	+	-	-

340	*Streptomyces* sp.	FB	-	+	-	-
341	*Ewingella* sp.	FB	+	-	-	~
343	*Burkholderia* sp.	FB	+	-	-	-
344	*Ewingella* sp.	FB	+	-	-	+
345	*Streptomyces* sp.	FB	-	+	-	-
346	*Streptomyces* sp.	FB	-	+	-	-
347	*Dermacoccus* sp.	R	-	-	-	+
348	*Enterobacter* sp.	FB	-	++	-	-
349	*Enterobacter* sp.	FB	-	-	-	+
350	*Enterobacter* sp.	FB	-	-	-	+
351	*Klebsiella* sp.	FB	-	-	-	+
352	*Klebsiella* sp.	FB	-	-	-	+
353	*Staphylococcus* sp.	R	-	+	-	-
355	*Staphylococcus* sp.	R	-	~	-	-
356	*Staphylococcus* sp.	R	-	+	-	-
357	*	R	-	+	-	+
358	*Staphylococcus* sp.	R	+	+	-	-
359	*Staphylococcus* sp.	R	+	+	-	~
360	*Staphylococcus* sp.	R	-	~	-	-
361	*Streptomyces* sp.	R	-	++	-	+
362	*Staphylococcus* sp.	R	-	~	-	-
363	*Dermacoccus* sp.	R	-	+	-	+
364	*Streptomyces* sp.	R	-	+	-	+
365	*Staphylococcus* sp.	R	-	~	-	-
366 a)	*	R	-	-	-	+
366 b)	*	R	+	+	+	+
367	*Bacillus* sp.	R	-	+	+	+
368	*Staphylococcus* sp.	R	-	~	-	-
369	*Bacillus* sp.	R	-	-	-	+
370	*Staphylococcus* sp.	R	-	~	-	-
371	*Streptomyces* sp.	R	-	+	-	+
372	*Staphylococcus* sp.	R	-	+	-	-
373	*Staphylococcus* sp.	R	-	+	-	-
374	*Paenisporosarcina* sp.	R	-	+	-	-
375	*Dermacoccus* sp.	R	-	+	-	++
376	*	R	-	+	-	-
377	*Streptomyces* sp.	R	-	+	-	+
378	*Staphylococcus* sp.	R	-	-	-	-
379	*Streptomyces* sp.	R	-	-	-	+
380	*Streptomyces* sp.	R	-	+	-	-
381	*Staphylococcus* sp.	R	-	~	-	+
382	*Staphylococcus* sp.	R	-	+	-	-
383	*Streptomyces* sp.	R	-	+	-	-
384	*Staphylococcus* sp.	R	-	~	-	~
385	*Staphylococcus* sp.	R	-	~	-	~

386	*Streptomyces* sp.	R	-	+	-	-
